# Supplementary material for: Single-cell and spatial transcriptomics enables probabilistic inference of cell type topography
Source: Commun Biol. 2020 Oct 9;3:565. doi: 10.1038/s42003-020-01247-y (PMC7547664; doi:10.1038/s42003-020-01247-y)
Supplement: Supplementary file 1 — Supplementary Information [file 42003_2020_1247_MOESM1_ESM.pdf]

Single-cell and spatial transcriptomics enables probabilistic inference of cell type topography

July 31, 2020

Supplementary Figures

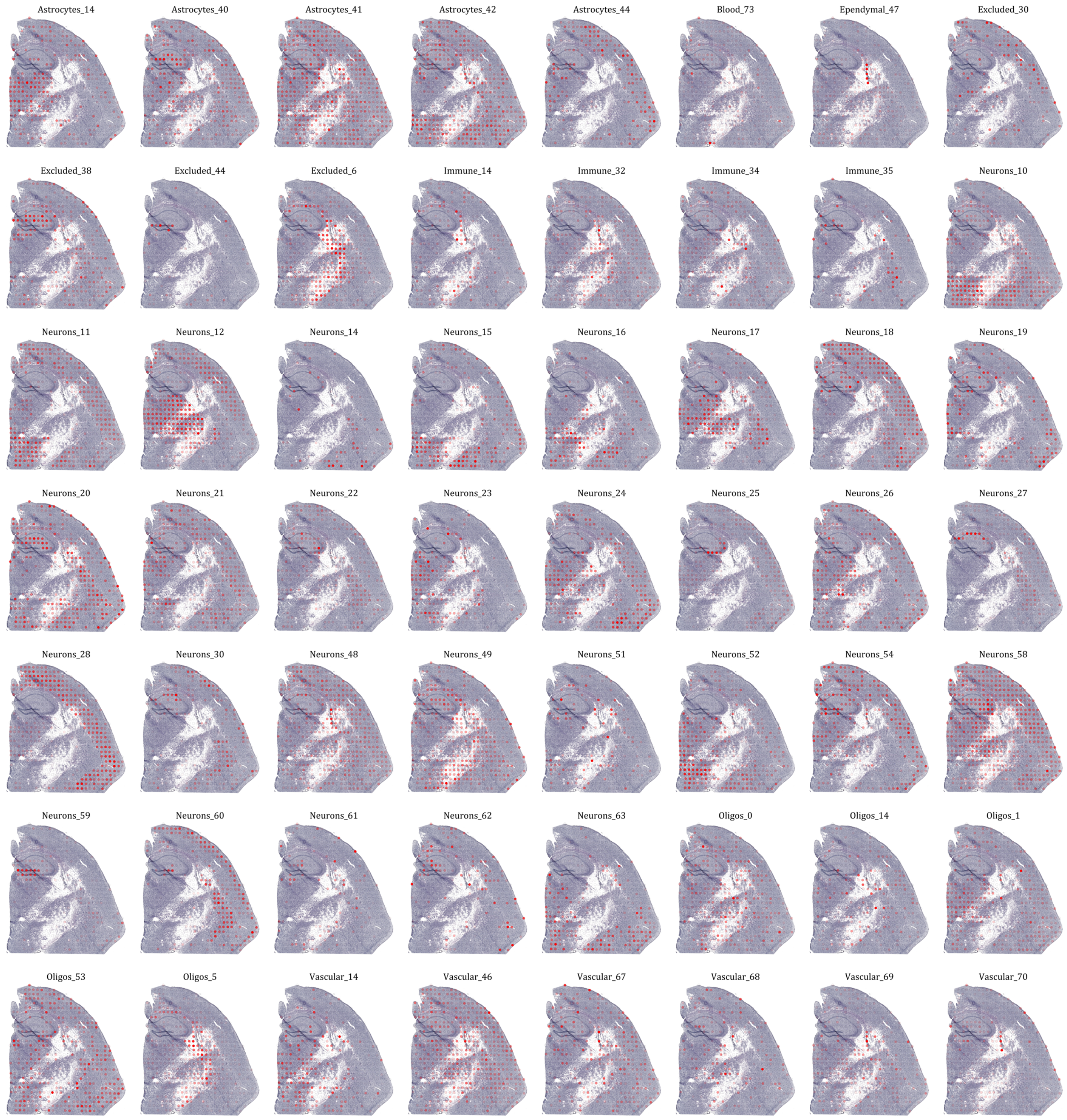

Supplementary Figure 1: Visualization of proportion estimates for section mb-ST1 (ST array, 100 micron spots) of the mouse brain, scaled within each cell type.

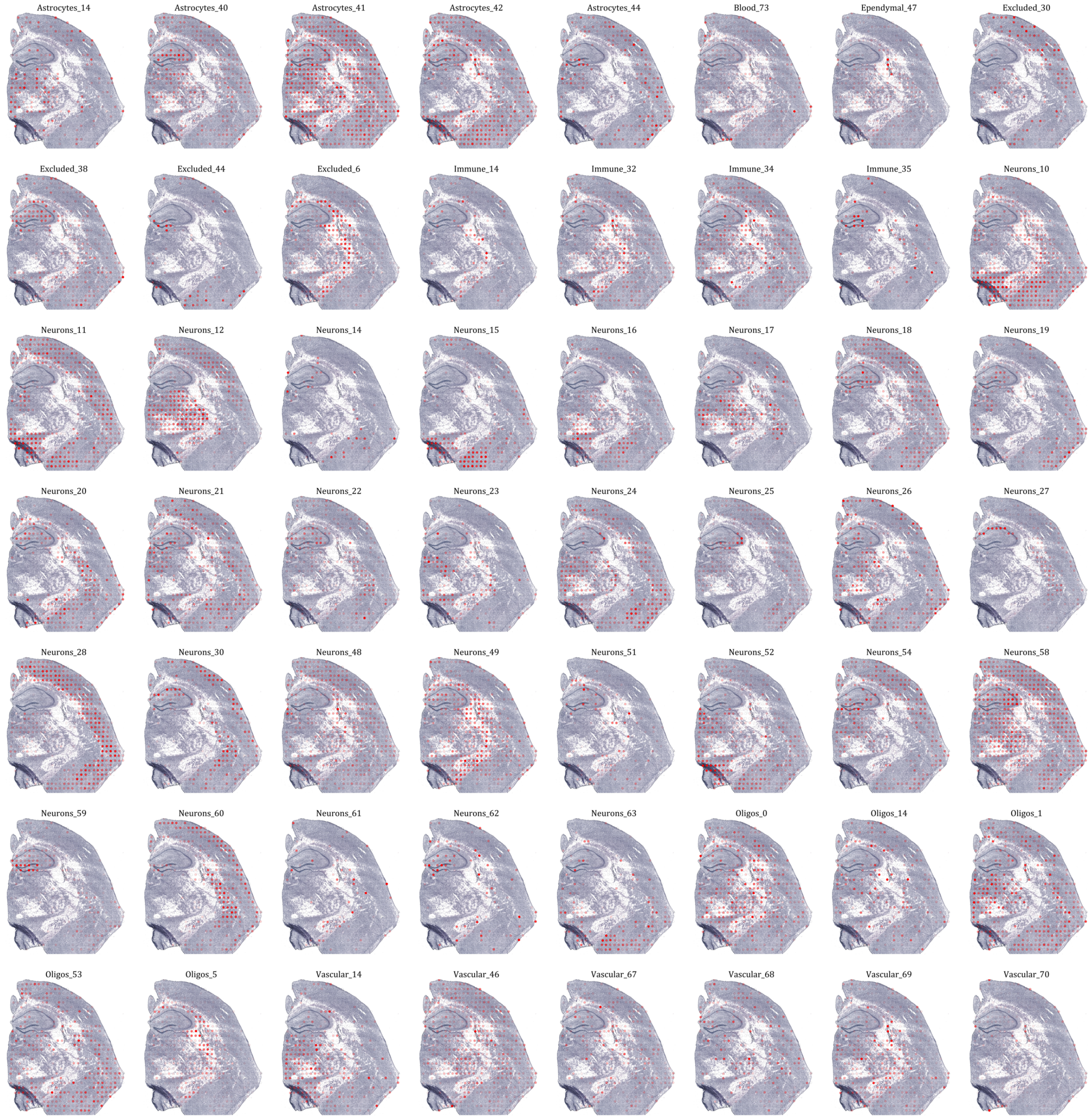

Supplementary Figure 2: Visualization of proportion estimates for section mb-ST2 (ST array, 100 micron spots) of the mouse brain, scaled within each cell type.

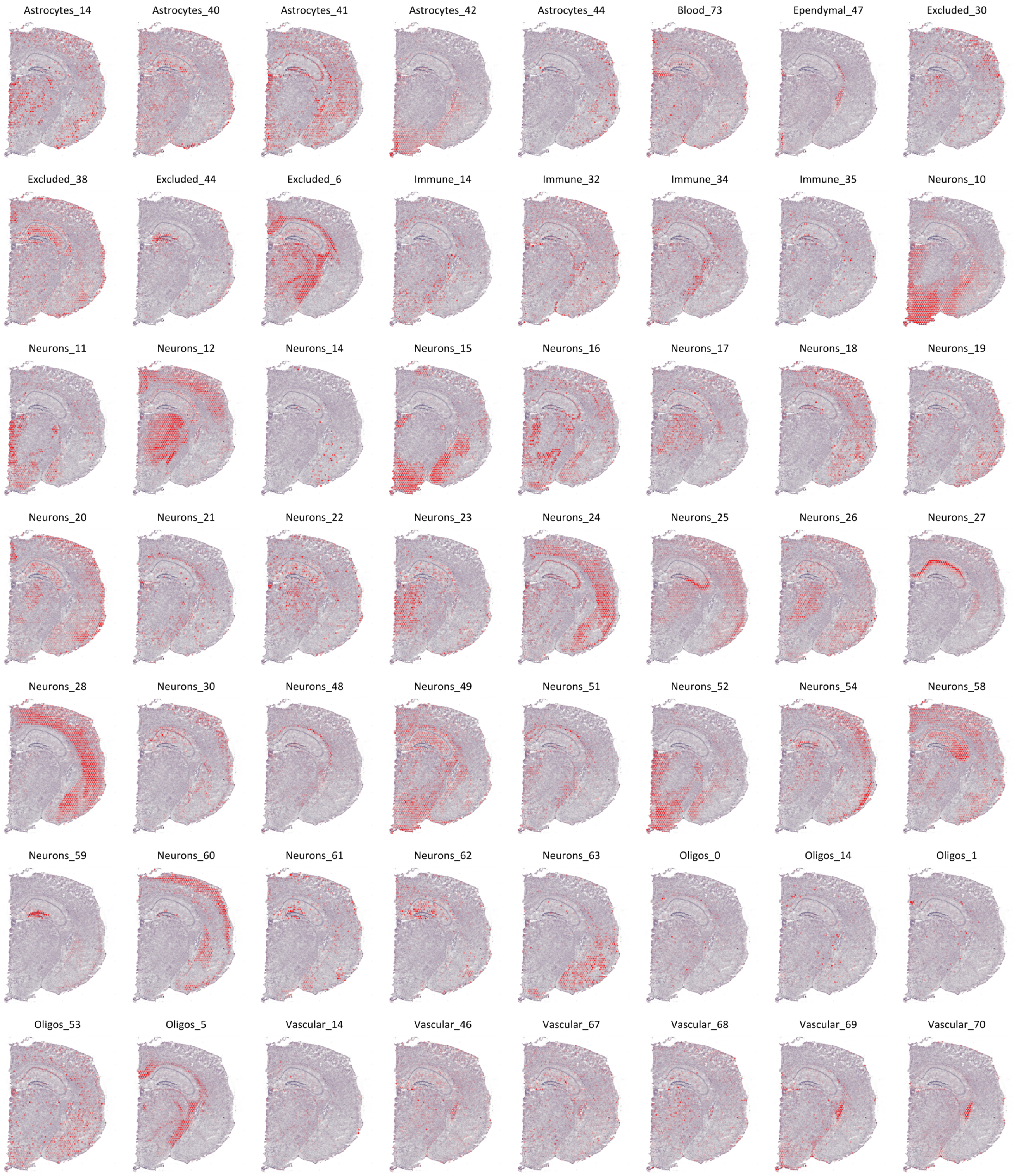

Supplementary Figure 3: Visualization of proportion estimates for section mb-V1 (Visium array, 55 micron spots), scaled within each cell type.

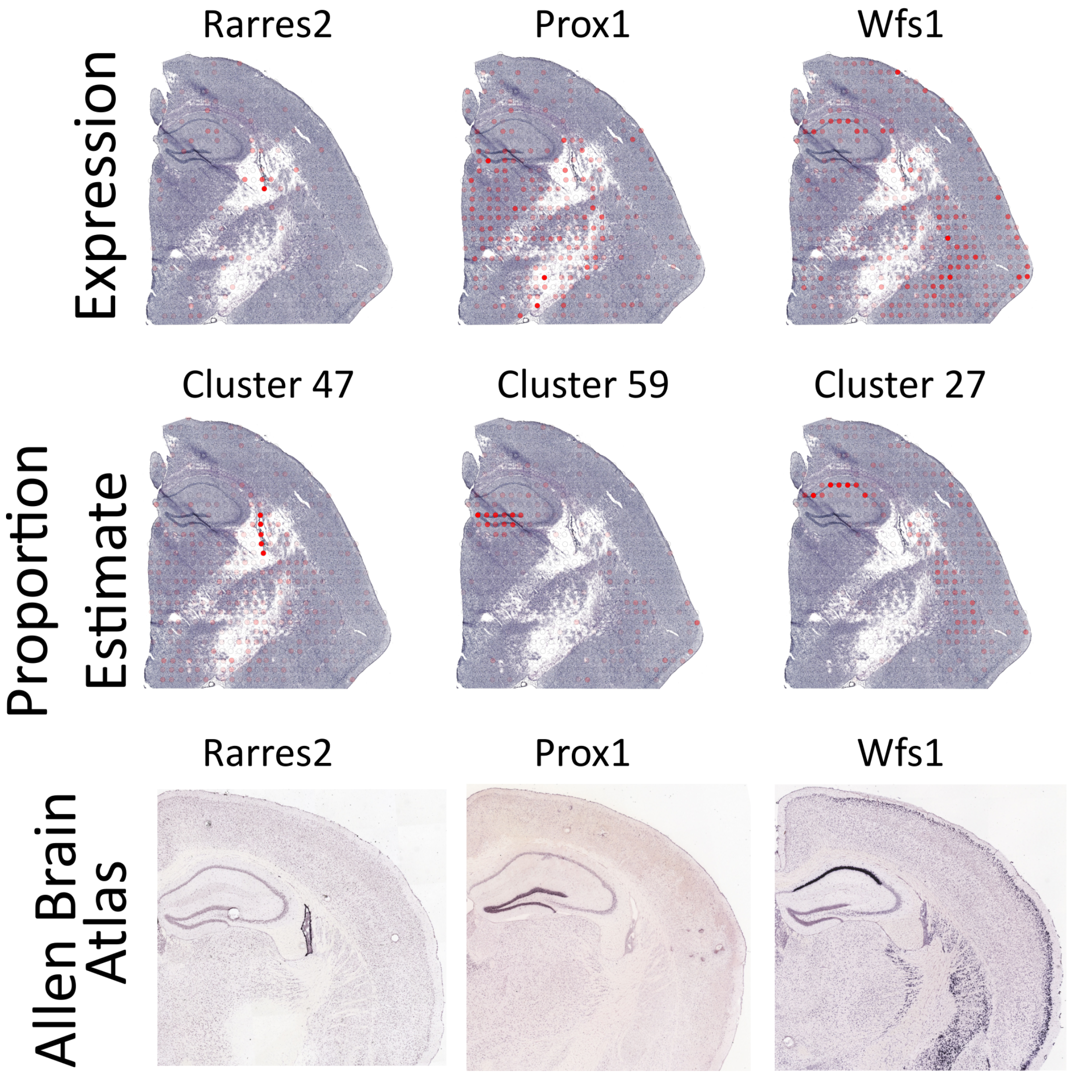

Supplementary Figure 4: Comparison between visualization of marker genes' relative expression (top) and proportion estimate (middle) in section mb-ST1, with Allen Brain Atlas ISH images (bottom) as reference. The relative gene expression is obtained by dividing the number of observed transcripts at a given spot (x_{sg}) by the total number of observed transcripts in the given spot. The relative gene expression values are visualized according to the same procedure as the proportion values (Methods).

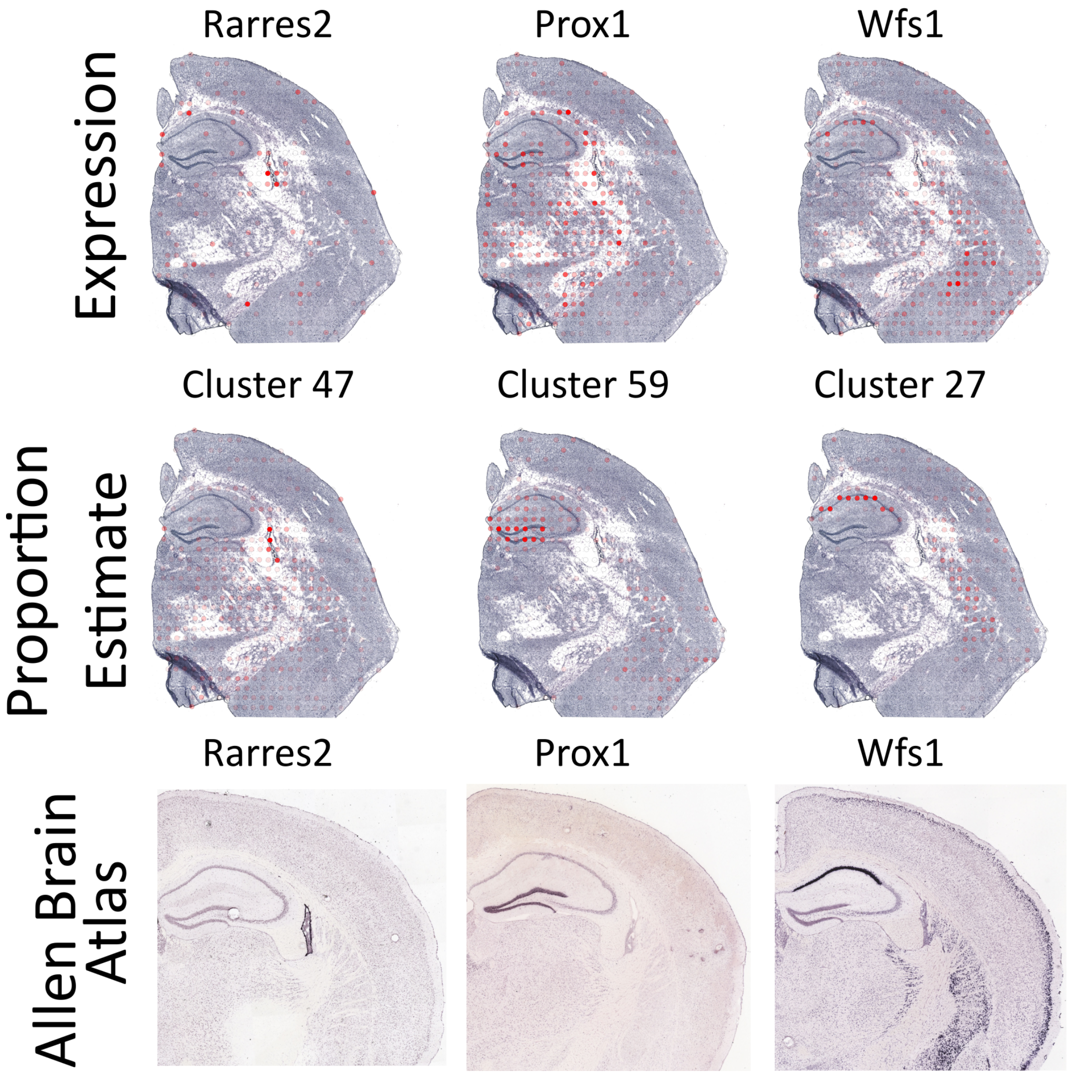

Supplementary Figure 5: Comparison between visualization of marker genes' relative expression (top) and proportion estimate (middle) in section mb-ST2, with Allen Brain Atlas ISH images (bottom) as reference. The relative gene expression is obtained by dividing the number of observed transcripts at a given spot (x_{sg}) by the total number of observed transcripts in the given spot. The relative gene expression values are visualized according to the same procedure as the proportion values (Methods).

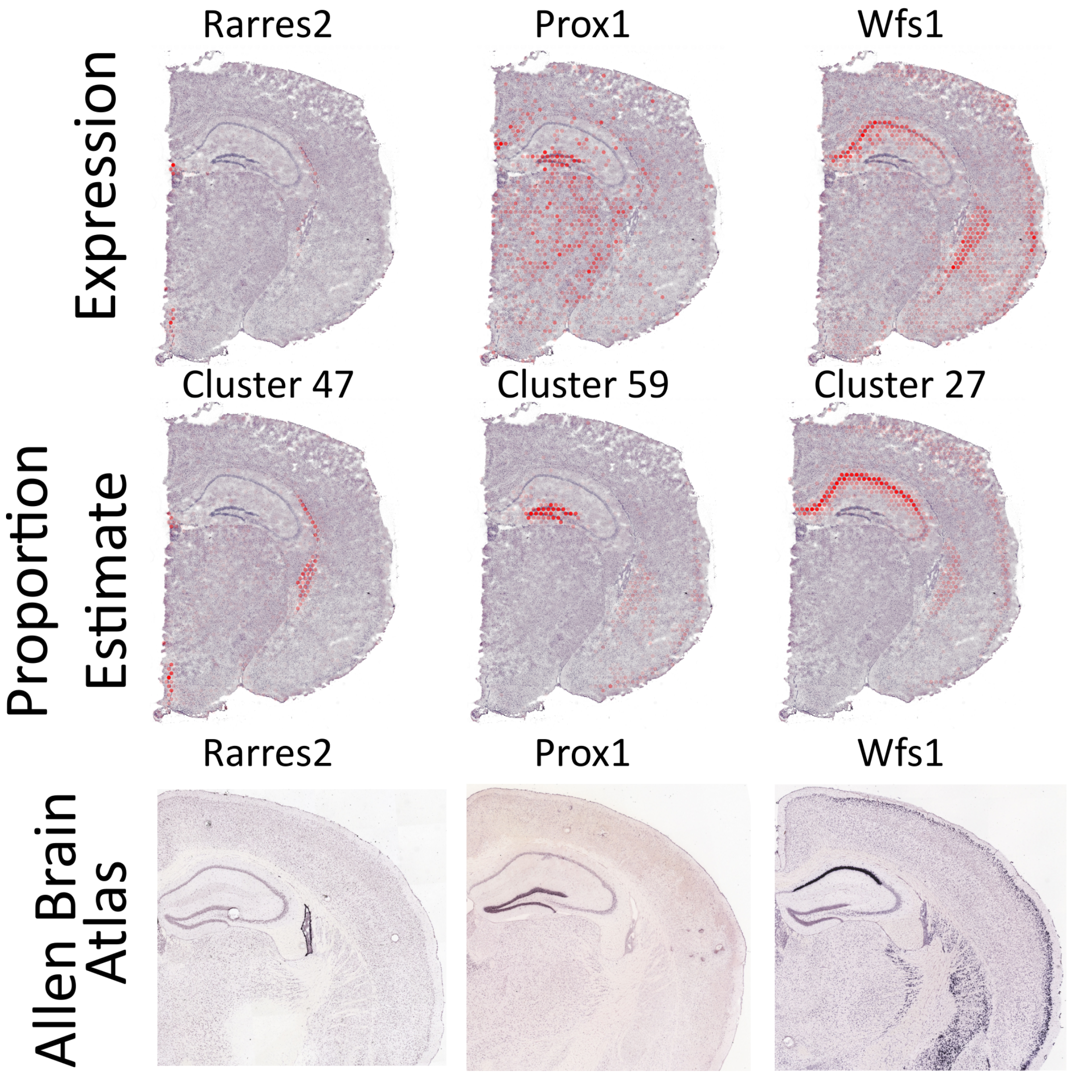

Supplementary Figure 6: Comparison between visualization of marker genes' relative expression (top) and proportion estimate (middle) in section mb-V1, with Allen Brain Atlas ISH images (bottom) as reference. The relative gene expression is obtained by dividing the number of observed transcripts at a given spot (x_{sg}) by the total number of observed transcripts in the given spot. The relative gene expression values are visualized according to the same procedure as the proportion values (Methods).

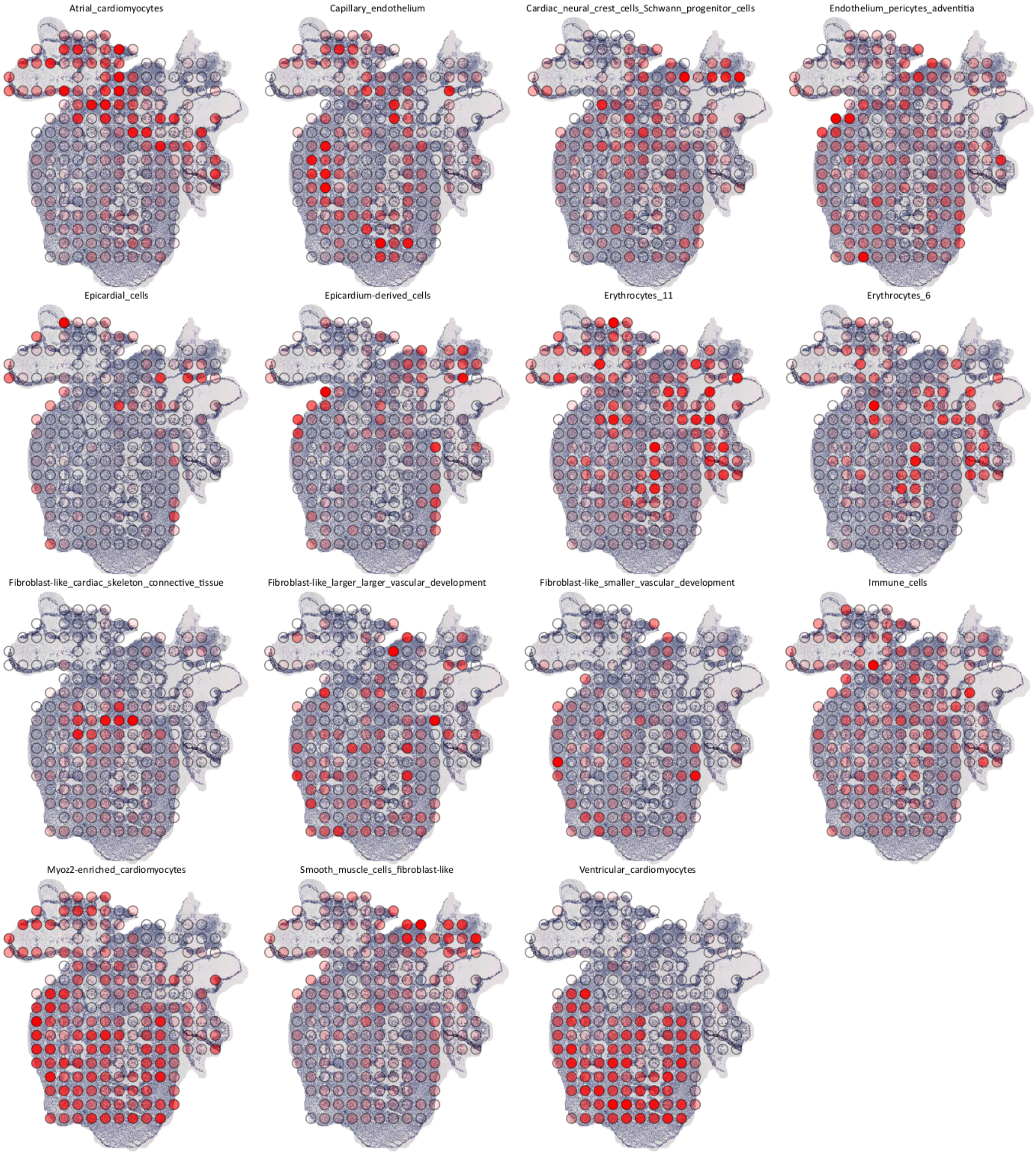

Supplementary Figure 7: Visualization of proportion estimates for section dh-A (from a series of eight independent sections from the same developmental heart, named A-H), scaled within each cell type.

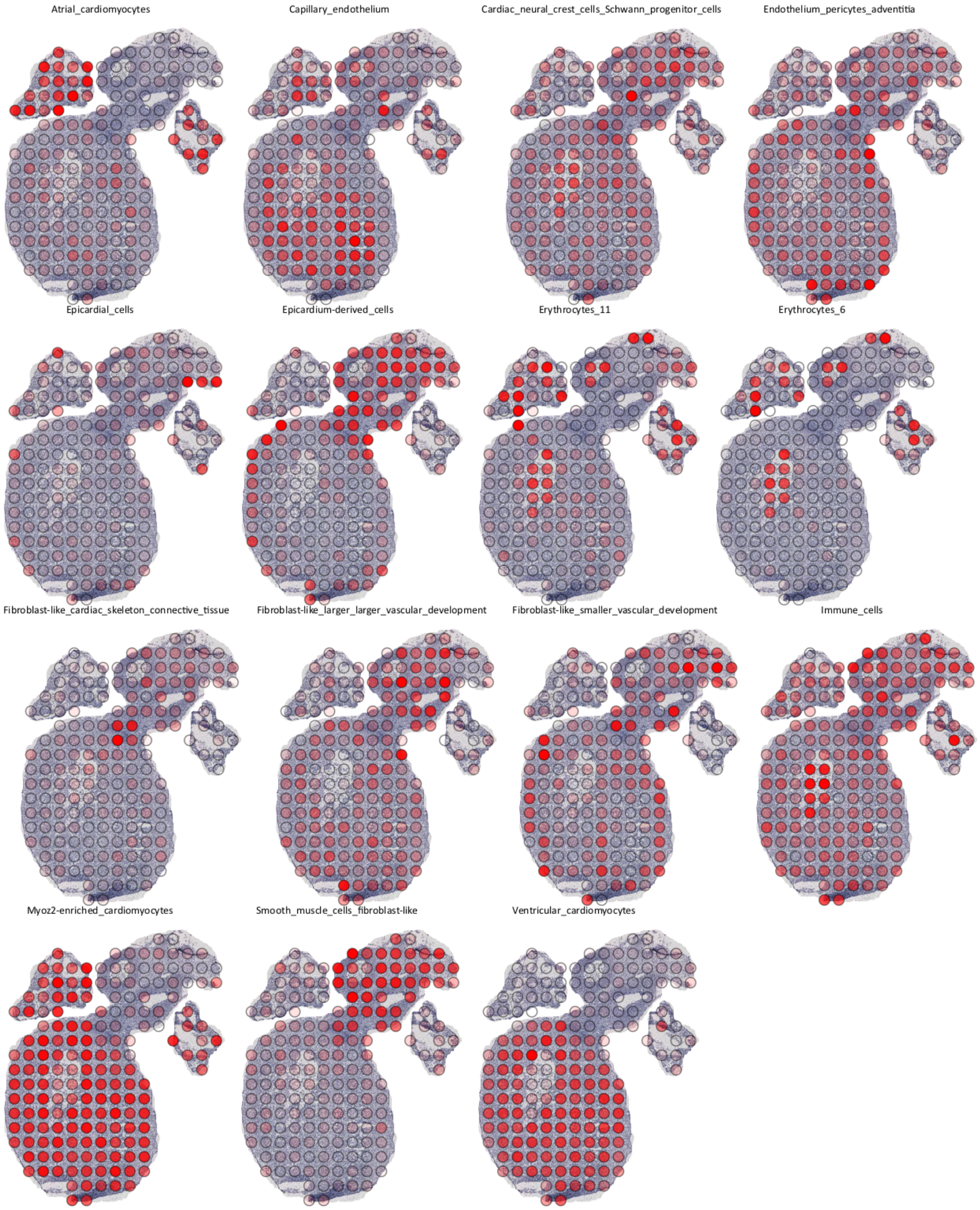

Supplementary Figure 8: Visualization of proportion estimates for section dh-B (from a series of eight independent sections from the same developmental heart, named A-H), scaled within each cell type.

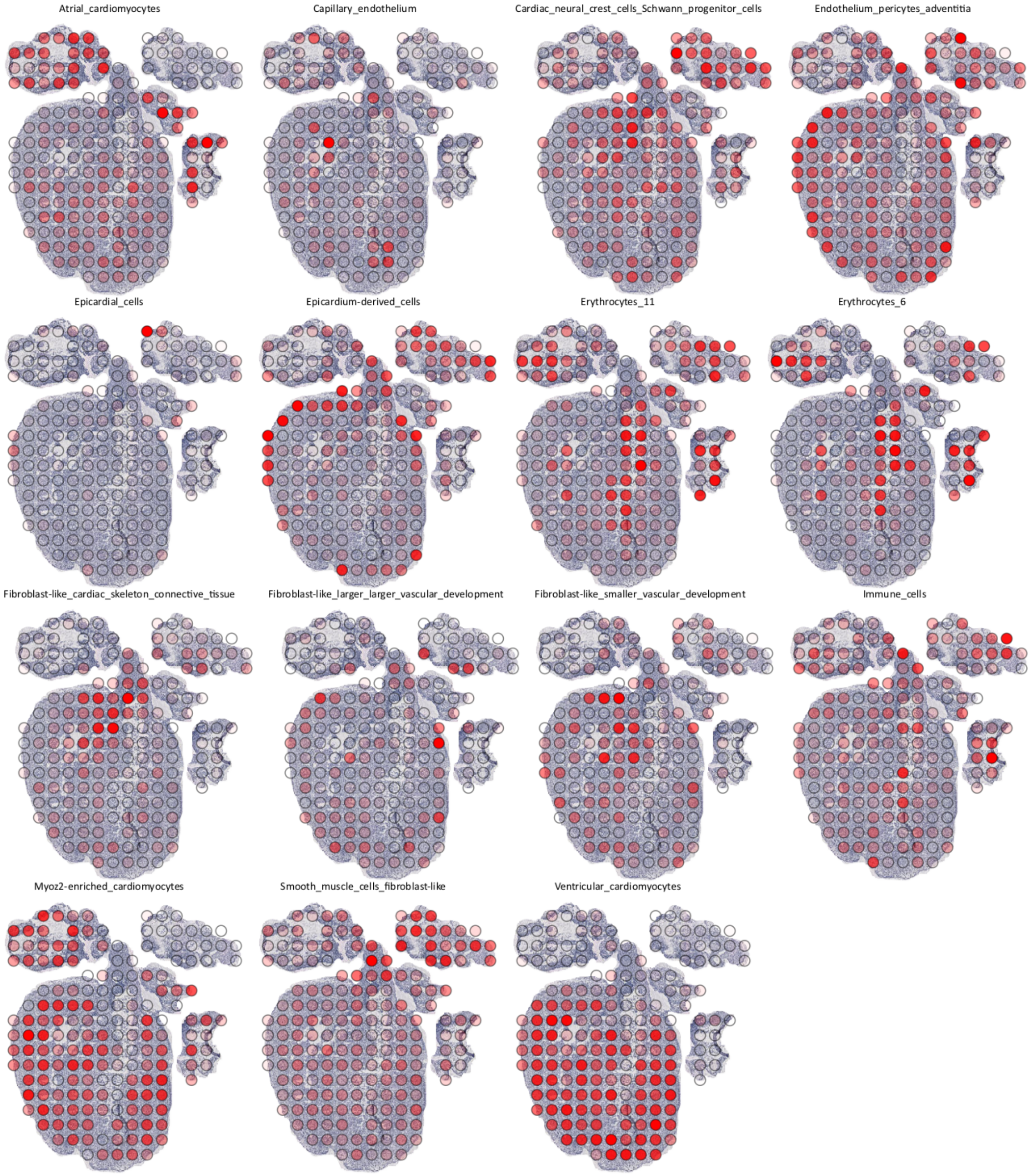

Supplementary Figure 9: Visualization of proportion estimates for section dh-C (from a series of eight independent sections from the same developmental heart, named A-H), scaled within each cell type.

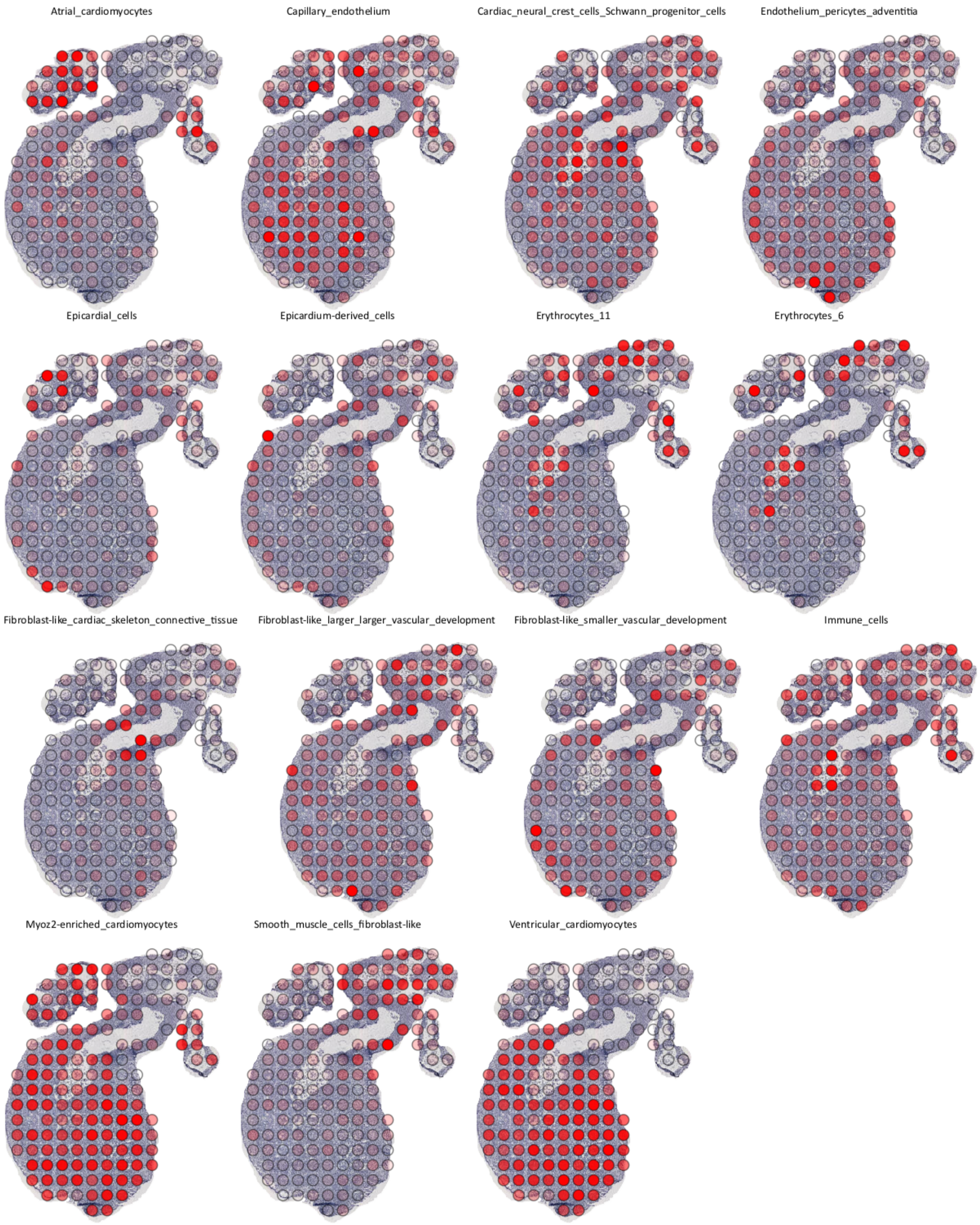

Supplementary Figure 10: Visualization of proportion estimates for section dh-D (from a series of eight independent sections from the same developmental heart, named A-H), scaled within each cell type.

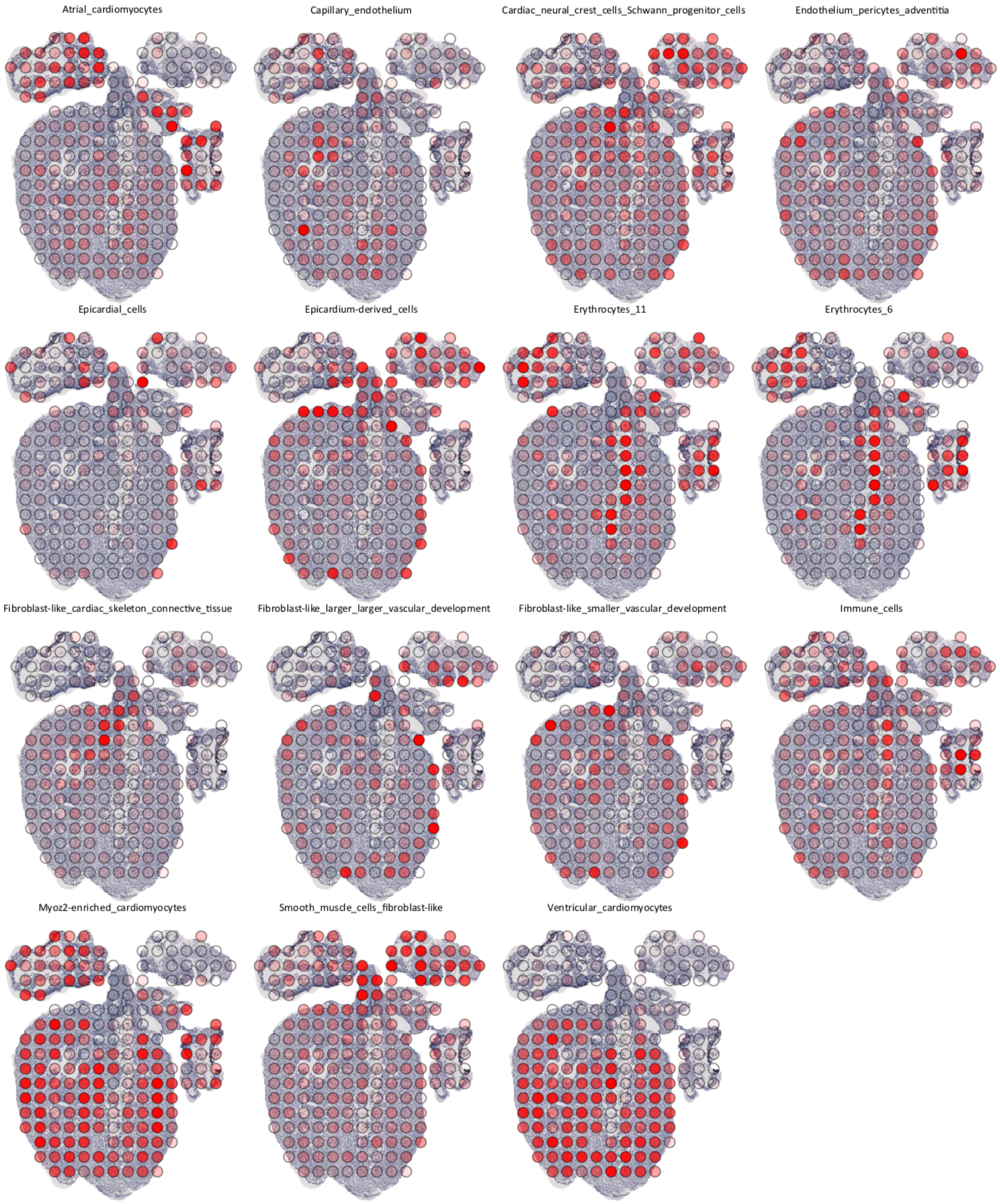

Supplementary Figure 11: Visualization of proportion estimates for section dh-E (from a series of eight independent sections from the same developmental heart, named A-H), scaled within each cell type.

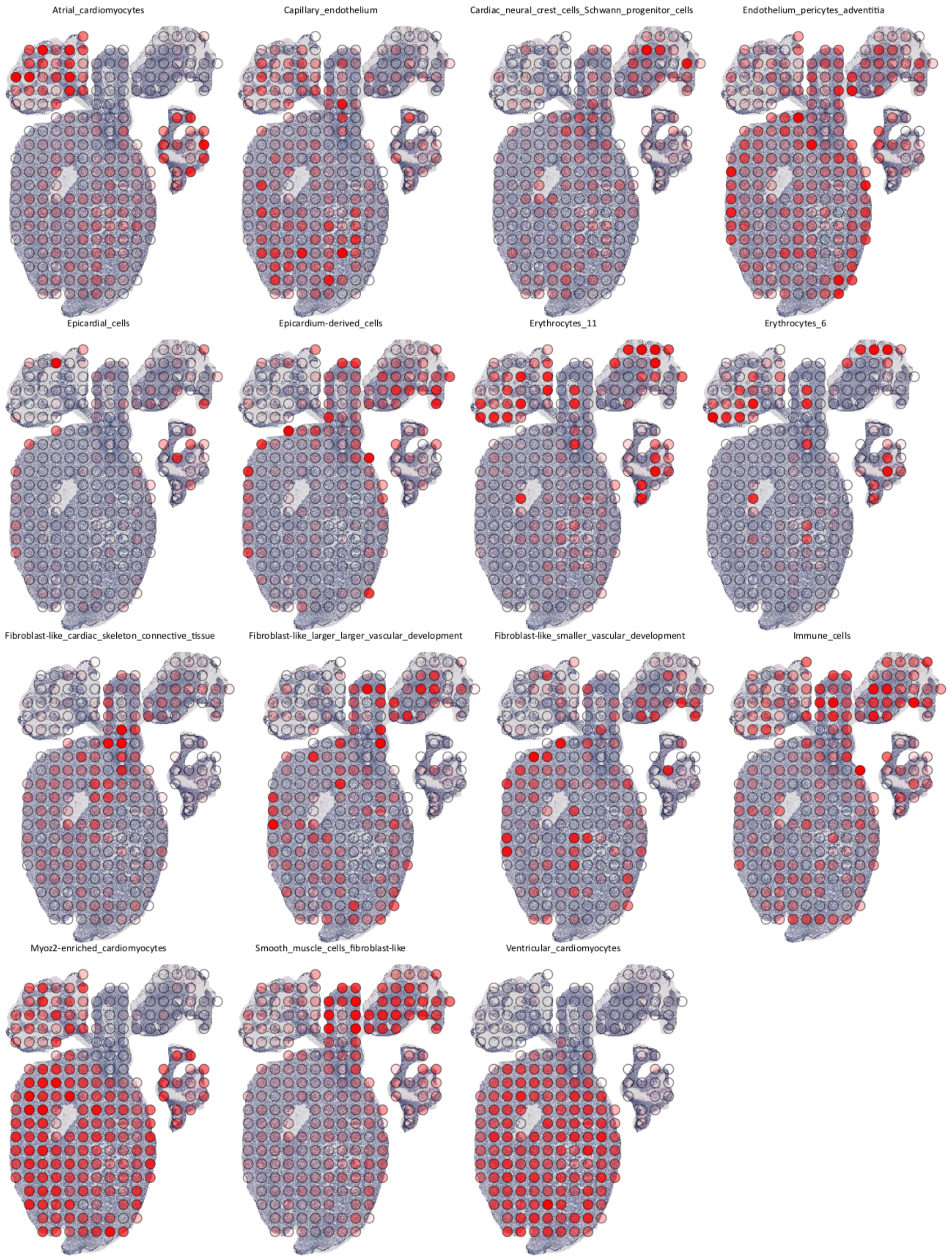

Supplementary Figure 12: Visualization of proportion estimates for section dh-F (from a series of eight independent sections from the same developmental heart, named A-H), scaled within each cell type.

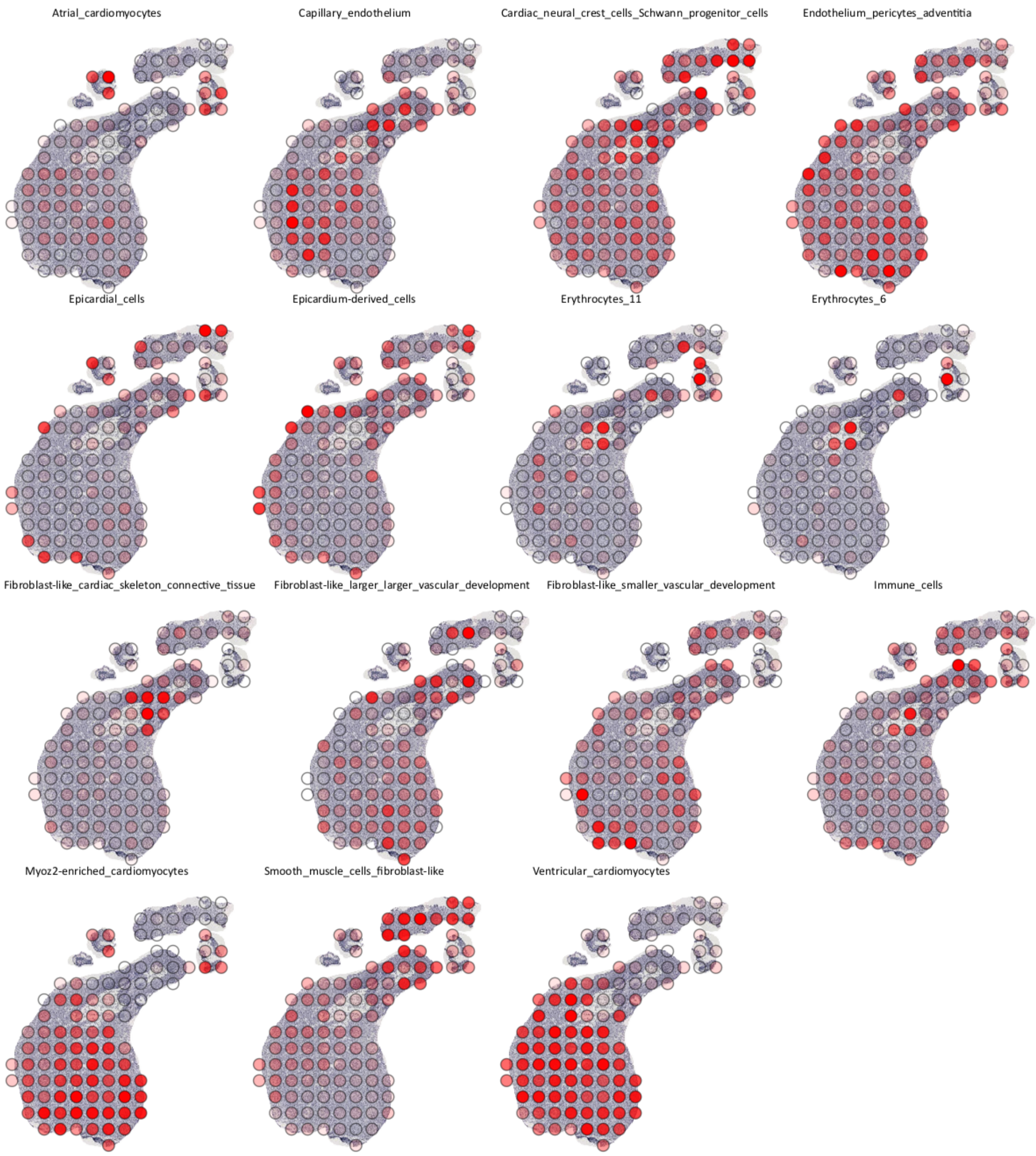

Supplementary Figure 13: Visualization of proportion estimates for section dh-G (from a series of eight independent sections from the same developmental heart, named A-H), scaled within each cell type.

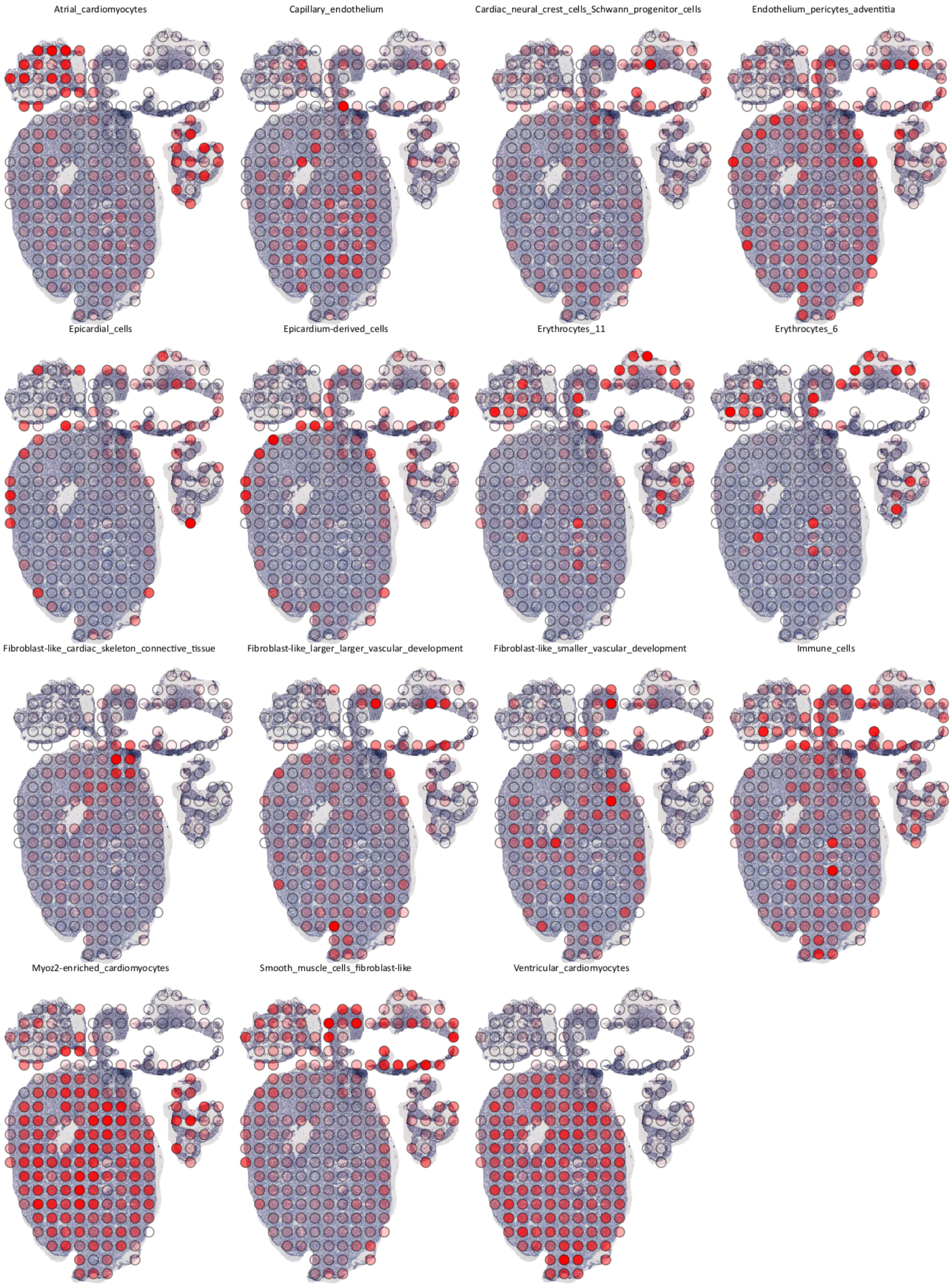

Supplementary Figure 14: Visualization of proportion estimates for section dh-H (from a series of eight independent sections from the same developmental heart, named A-H), scaled within each cell type.

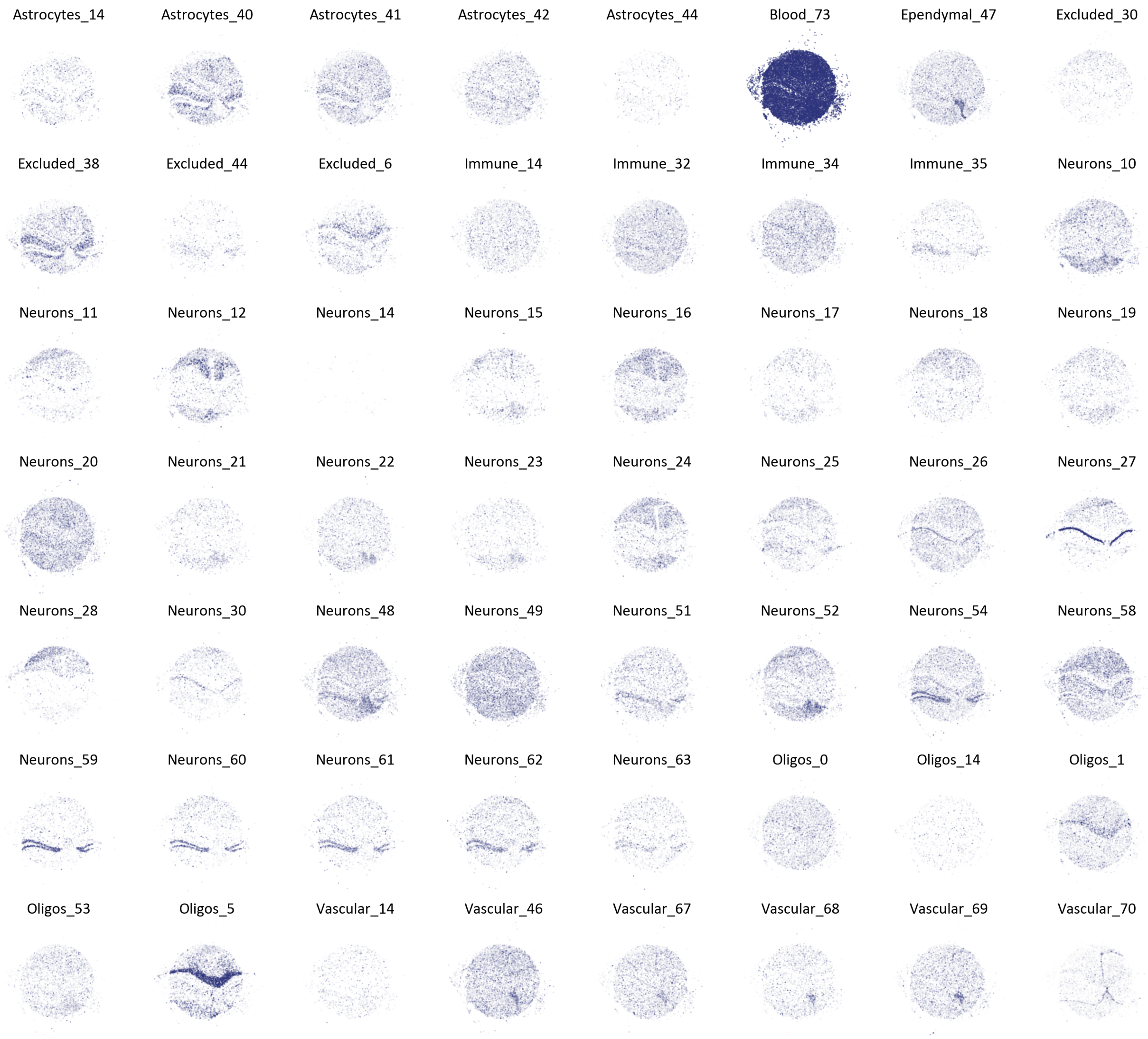

Supplementary Figure 15: Visualization of the proportion estimates for the Slide-seq mouse brain hippocampus data. The alpha value is proportional to the estimated proportion value, meaning that dark blue areas correspond to regions where the given cell type constitutes a high proportion of the cells while the opposite is true for those of white color.

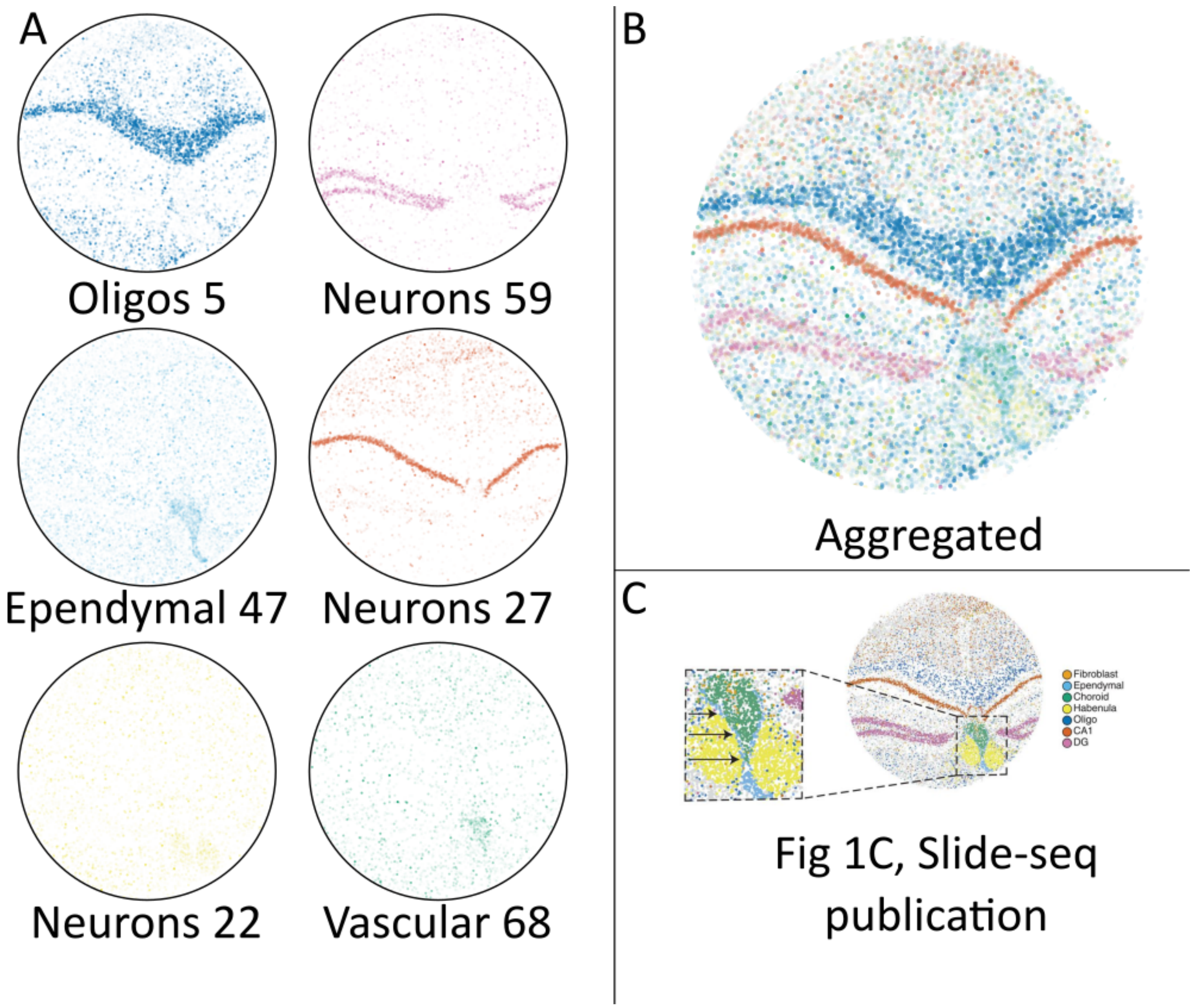

Supplementary Figure 16: **A)** Visualization of proportion estimates for six clusters (Oligos 5, Neurons 59, Ependymal 47, Neurons 27, Neurons 22 and Vascular 68) present in the single cell data. These clusters are colored using the same palette as the corresponding cell types in the Slide-seq method paper. [1] **B)** Aggregation of the six types visualized in A, produced by plotting their respective proportion values in one single plot. **C)** Modified version of Fig. 1C from the Slide-seq method paper. [1]

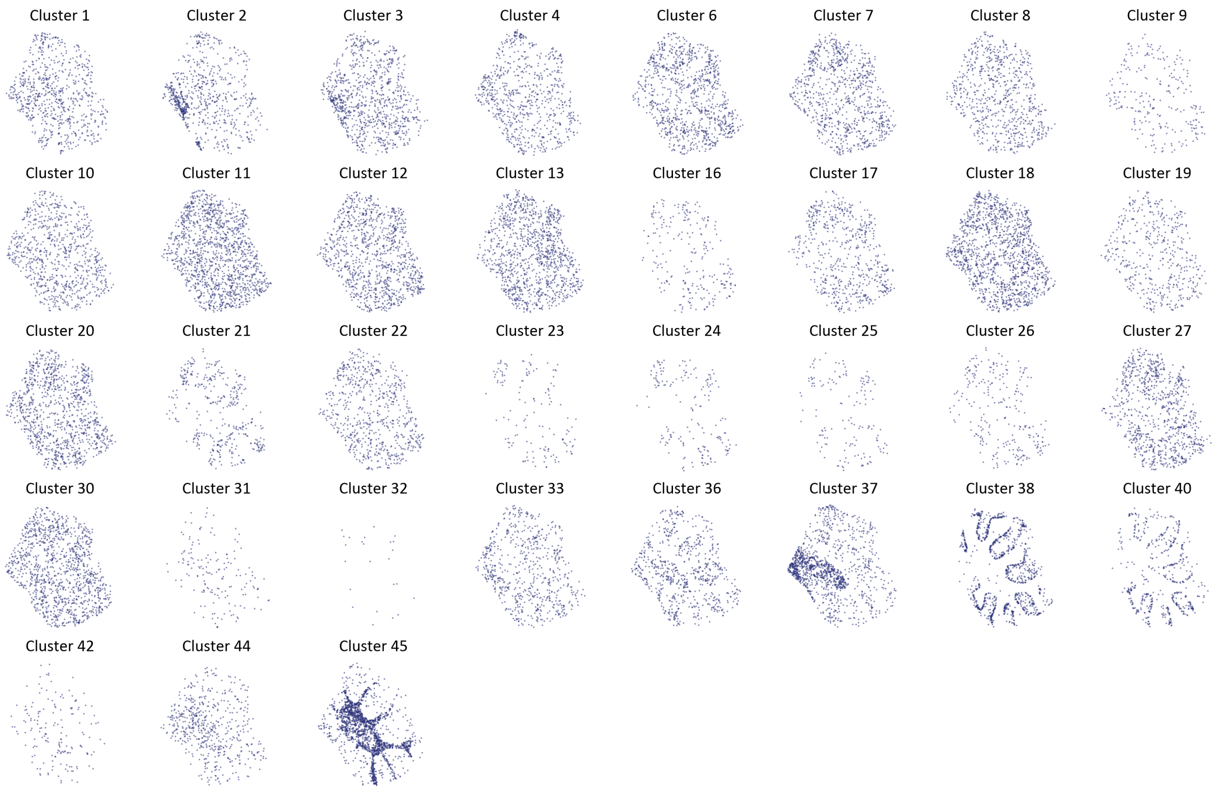

Supplementary Figure 17: Visualization of the proportion estimates for the Slide-seq mouse brain cerebellum data. Visualized by the same procedure as outlined in Figure 15.

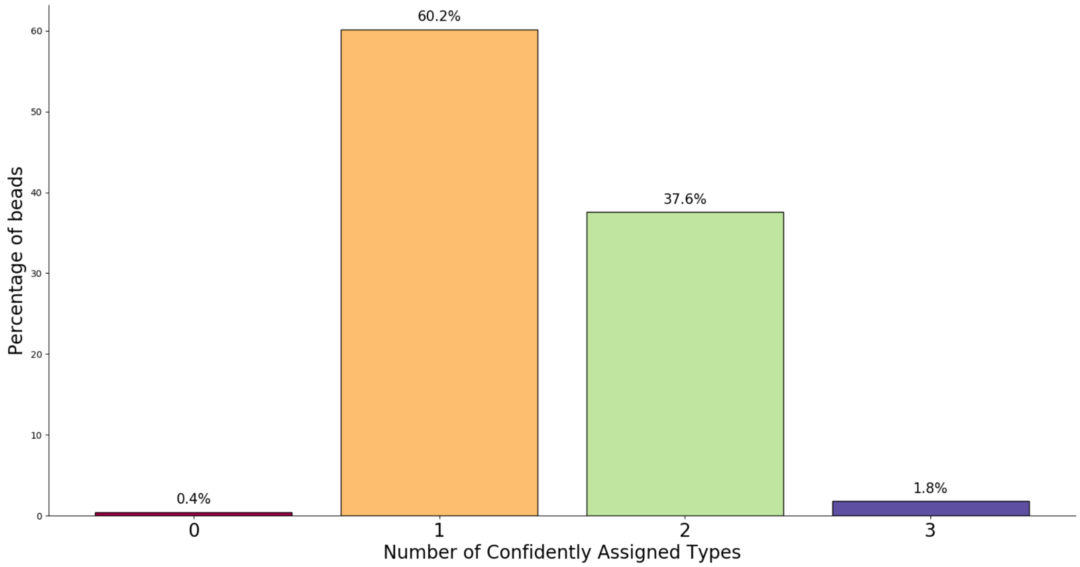

Supplementary Figure 18: Number of cell types confidently assigned to each bead in the mouse brain cerbellum data set. Confidently cell types are those (within a given bead) with a proportion value higher than half the L2-norm of the associated proportion vector, see Supplementary Section 1.6 for further details.

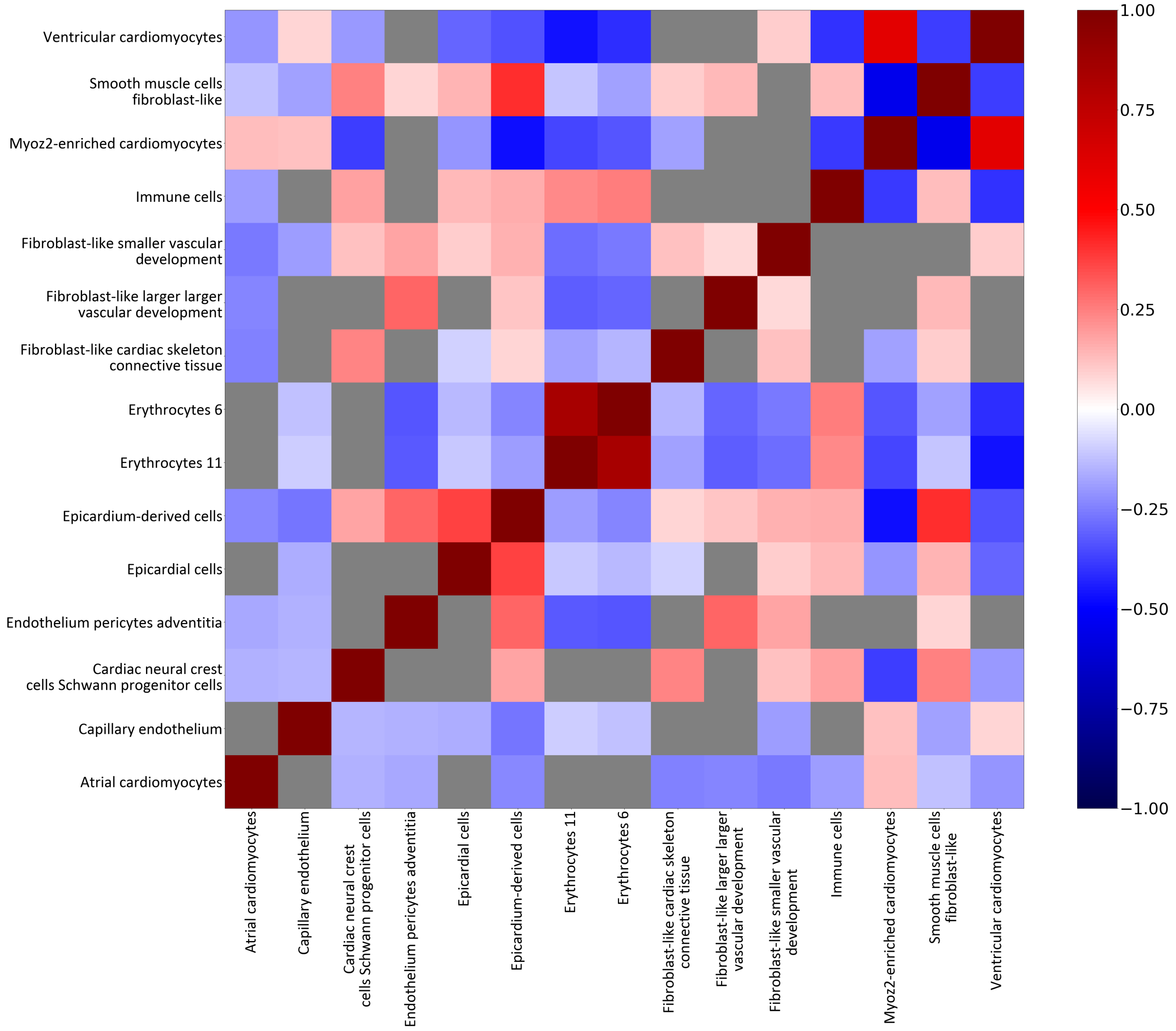

Supplementary Figure 19: Correlation between cell types (Methods) within the developmental heart. Gray areas represent correlation values where the correlation is not significant ($p \leq 0.01$). The correlation values are computed over all 8 sections ($n = 1375$ capture locations).

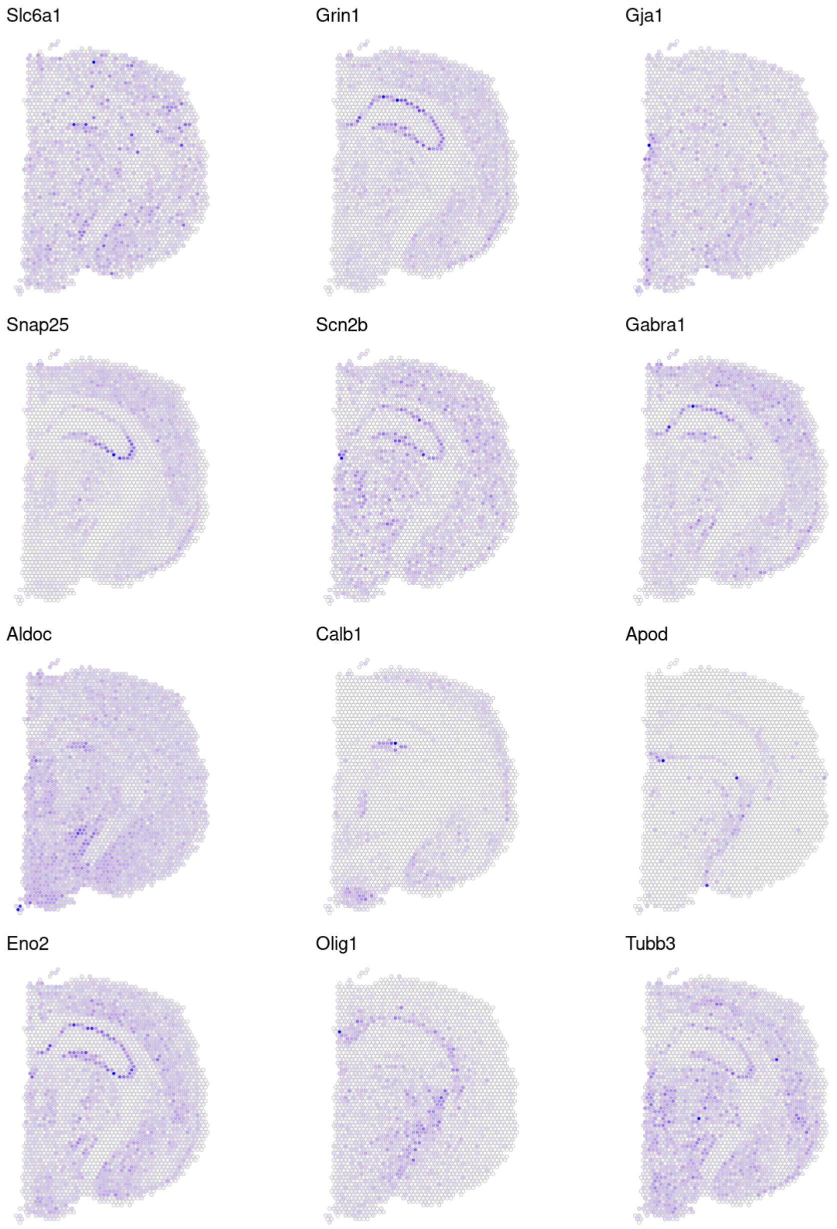

Supplementary Figure 20: In the mb-V1 sample; spatial gene expression of 12 genes listed, by the database panglaodb.se, as markers for different cell types found within the mouse brain [2]

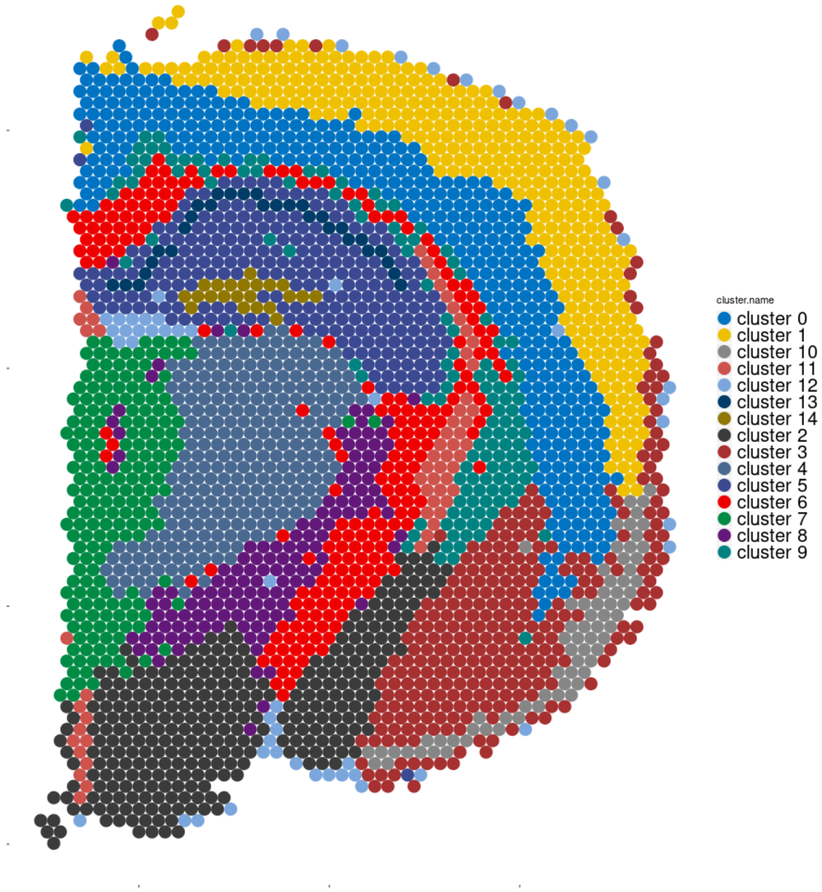

Supplementary Figure 21: Expression-based clustering of mb-V1, the normalization and clustering was obtained by using Seurat (v.3.0). A total of 15 clusters were obtained using a resolution of 0.8 in the SNN (Shared Nearest Neighbor) approach. Capture locations belonging to the same cluster share the same color. For more details, see Supplementary Section 1.1.1

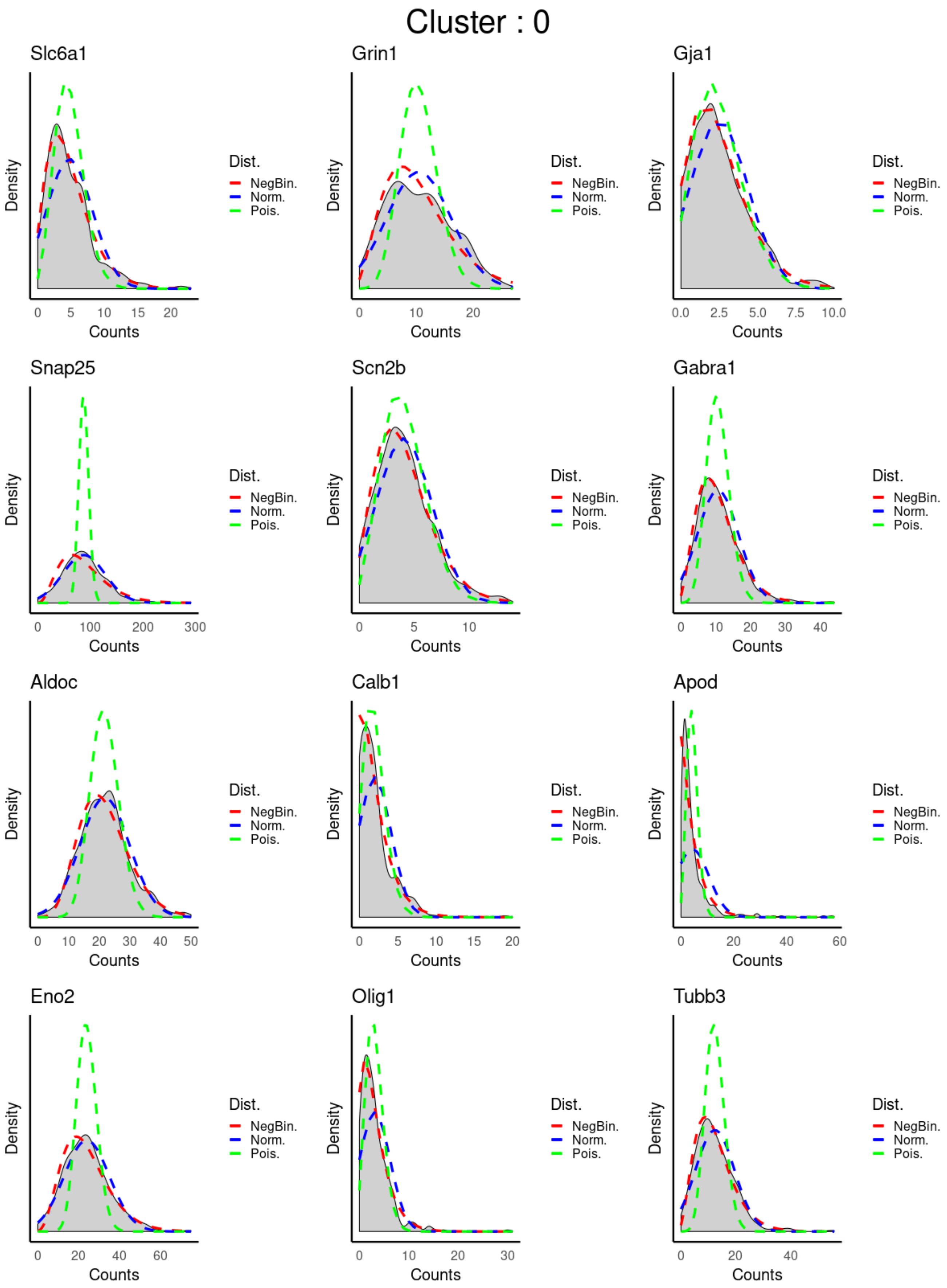

Supplementary Figure 22: Fitted vs. Empirical Distributions for Cluster 0. Gray - empirical distribution, Red - Negative Binomial, Green - Poisson, Blue - Normal

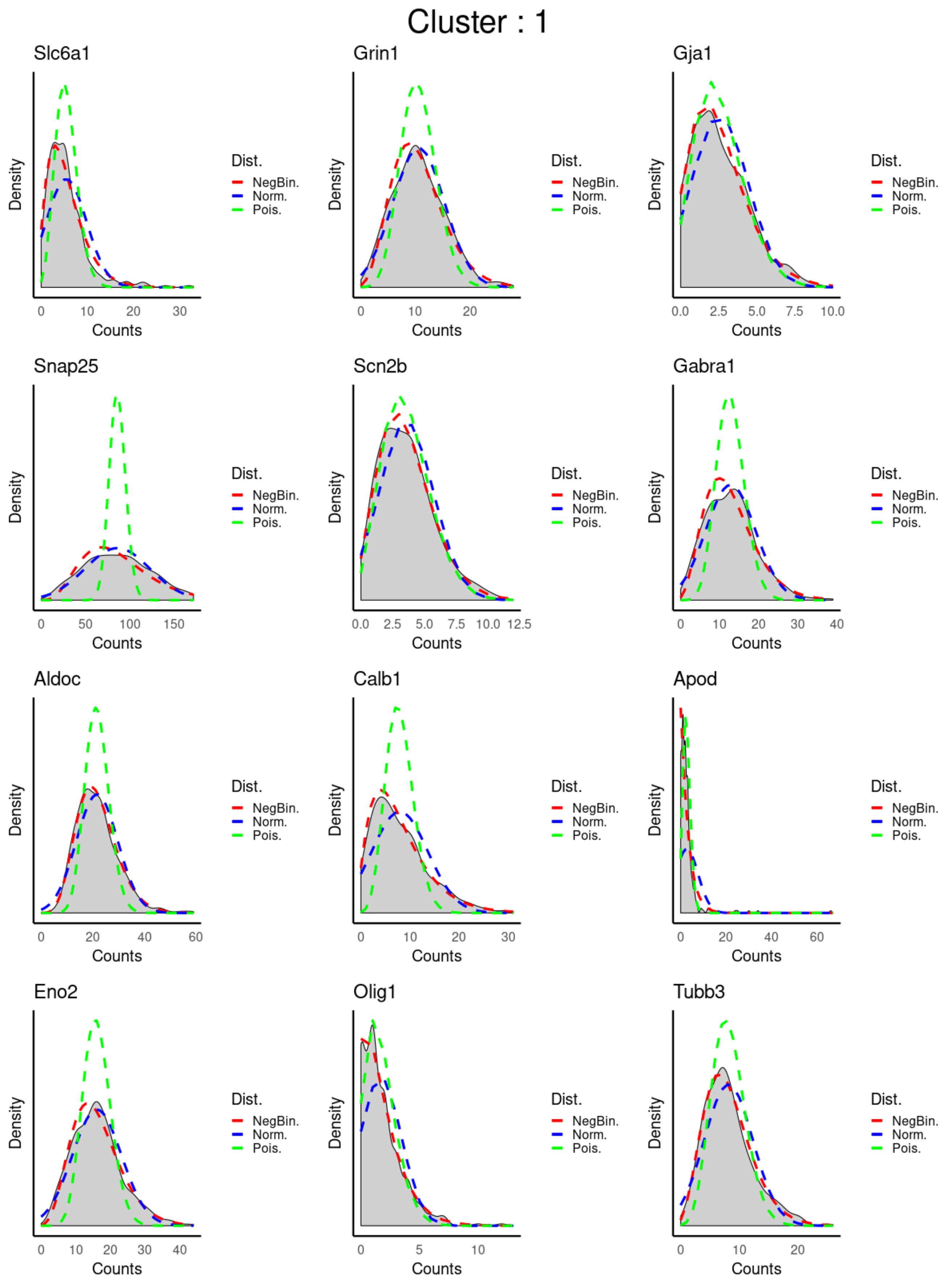

Supplementary Figure 23: Fitted vs. Empirical Distributions for Cluster 1. Gray - empirical distribution, Red - Negative Binomial, Green - Poisson, Blue - Normal

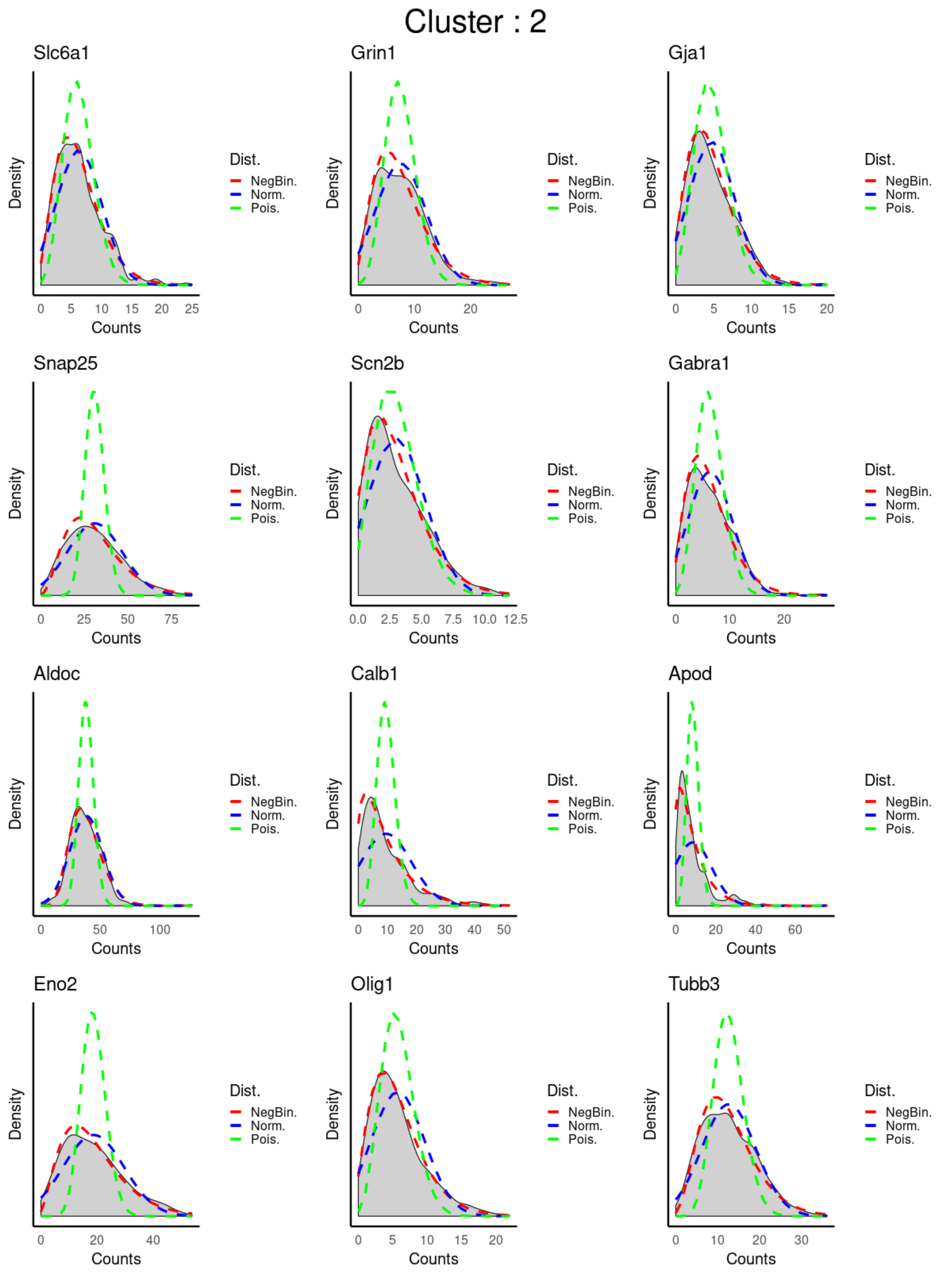

Supplementary Figure 24: Fitted vs. Empirical Distributions for Cluster 2. Gray - empirical distribution, Red - Negative Binomial, Green - Poisson, Blue - Normal

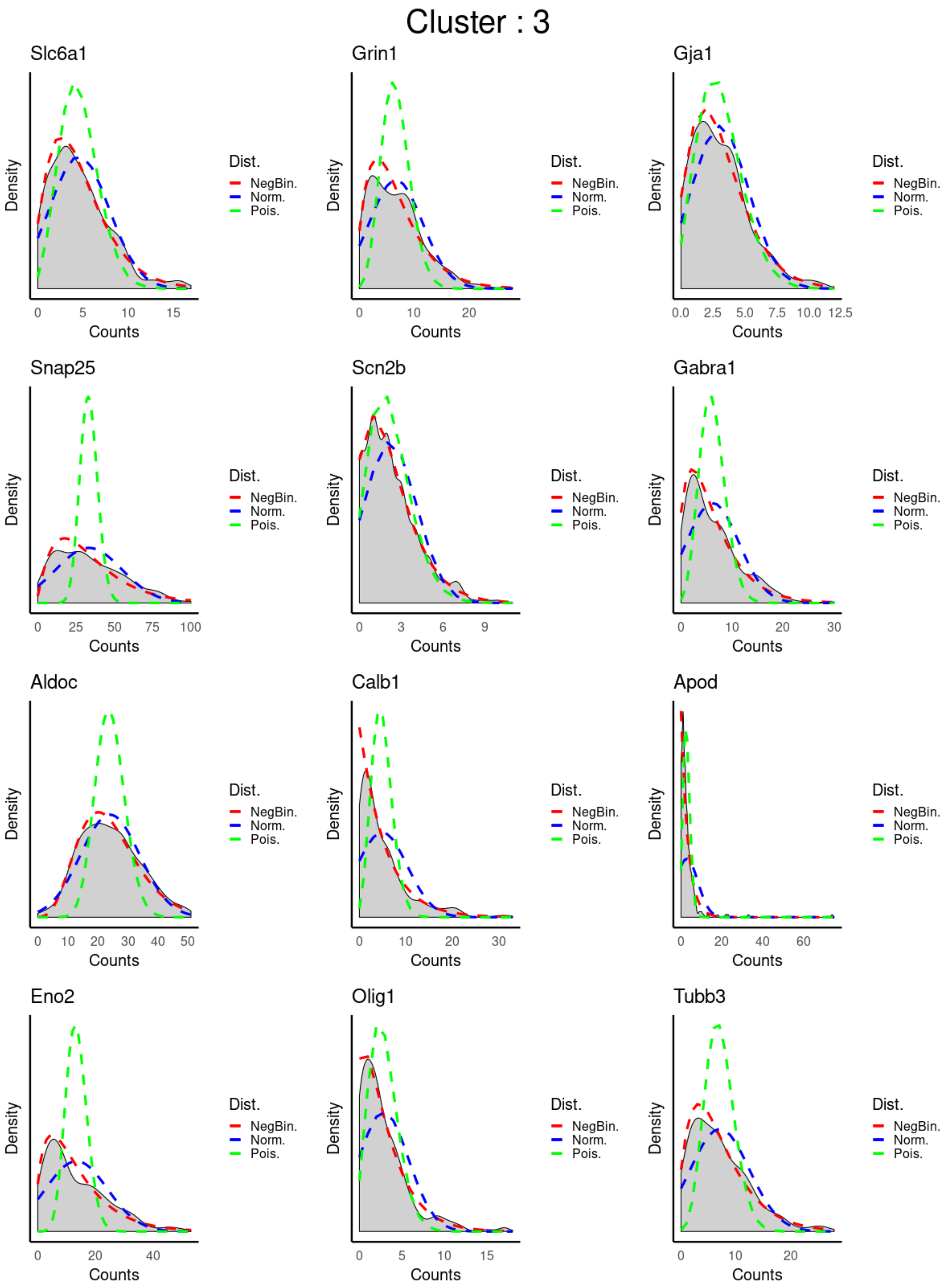

Supplementary Figure 25: Fitted vs. Empirical Distributions for Cluster 3. Gray - empirical distribution, Red - Negative Binomial, Green - Poisson, Blue - Normal

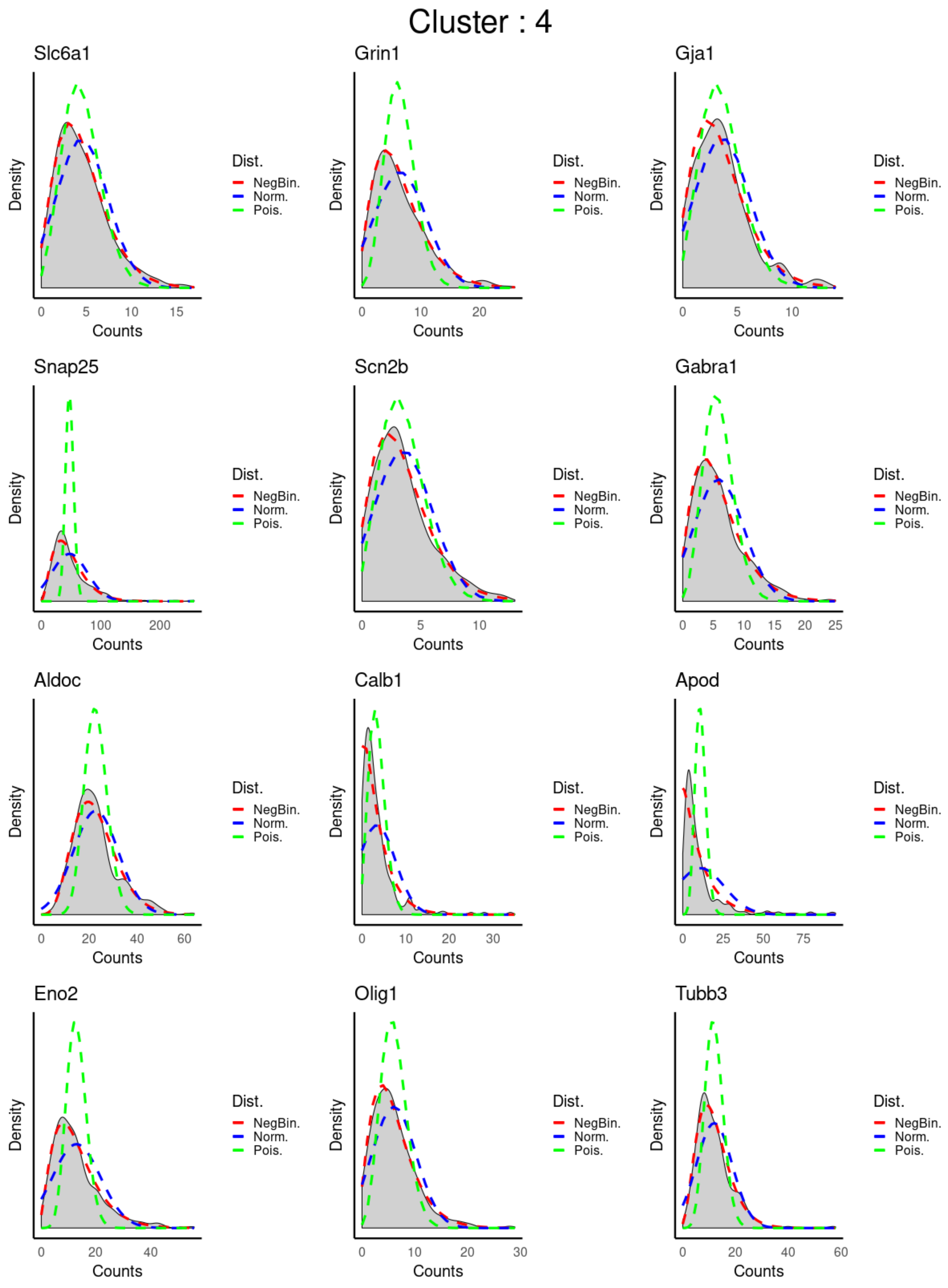

Supplementary Figure 26: Fitted vs. Empirical Distributions for Cluster 4. Gray - empirical distribution, Red - Negative Binomial, Green - Poisson, Blue - Normal

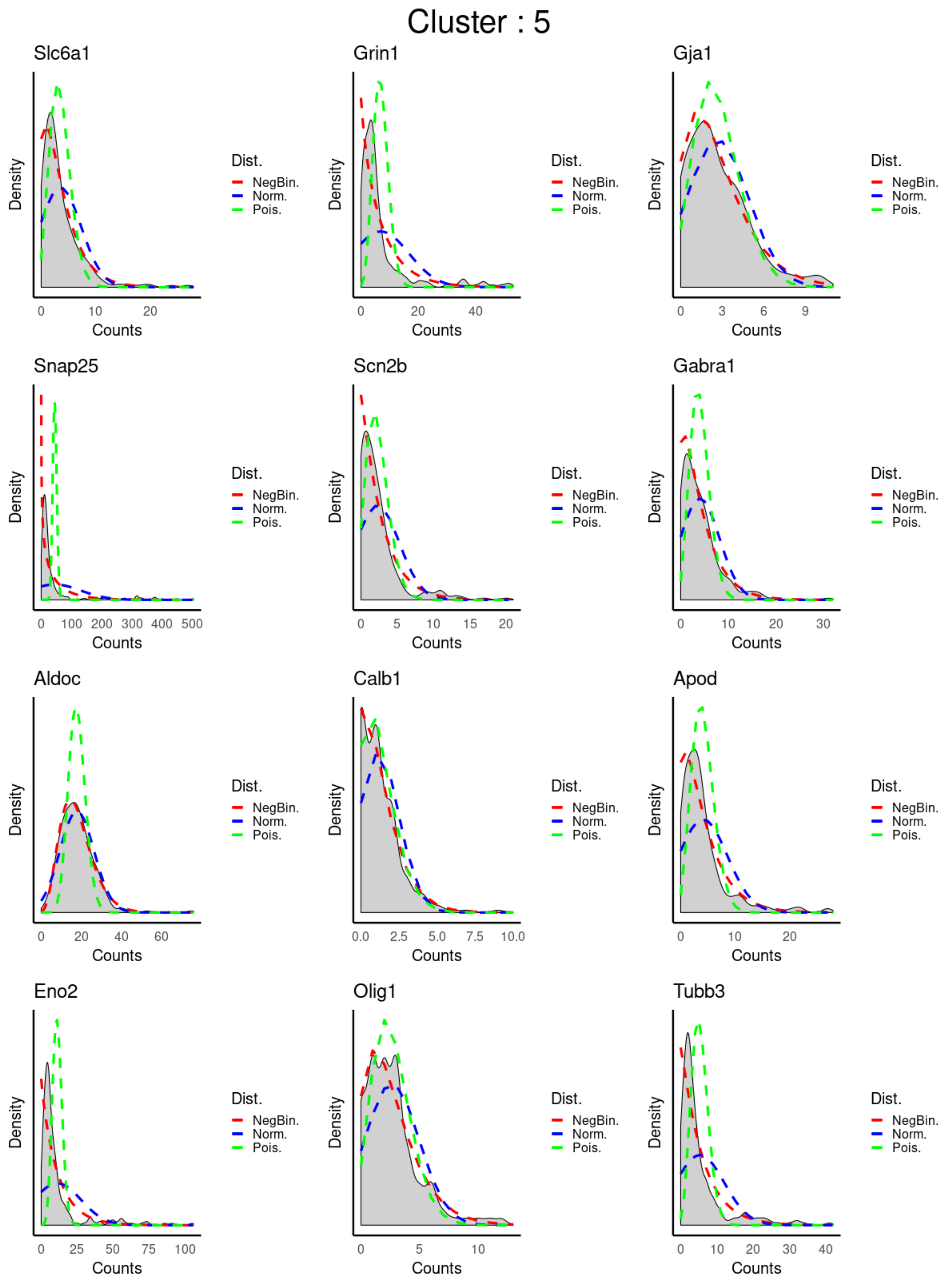

Supplementary Figure 27: Fitted vs. Empirical Distributions for Cluster 5. Gray - empirical distribution, Red - Negative Binomial, Green - Poisson, Blue - Normal

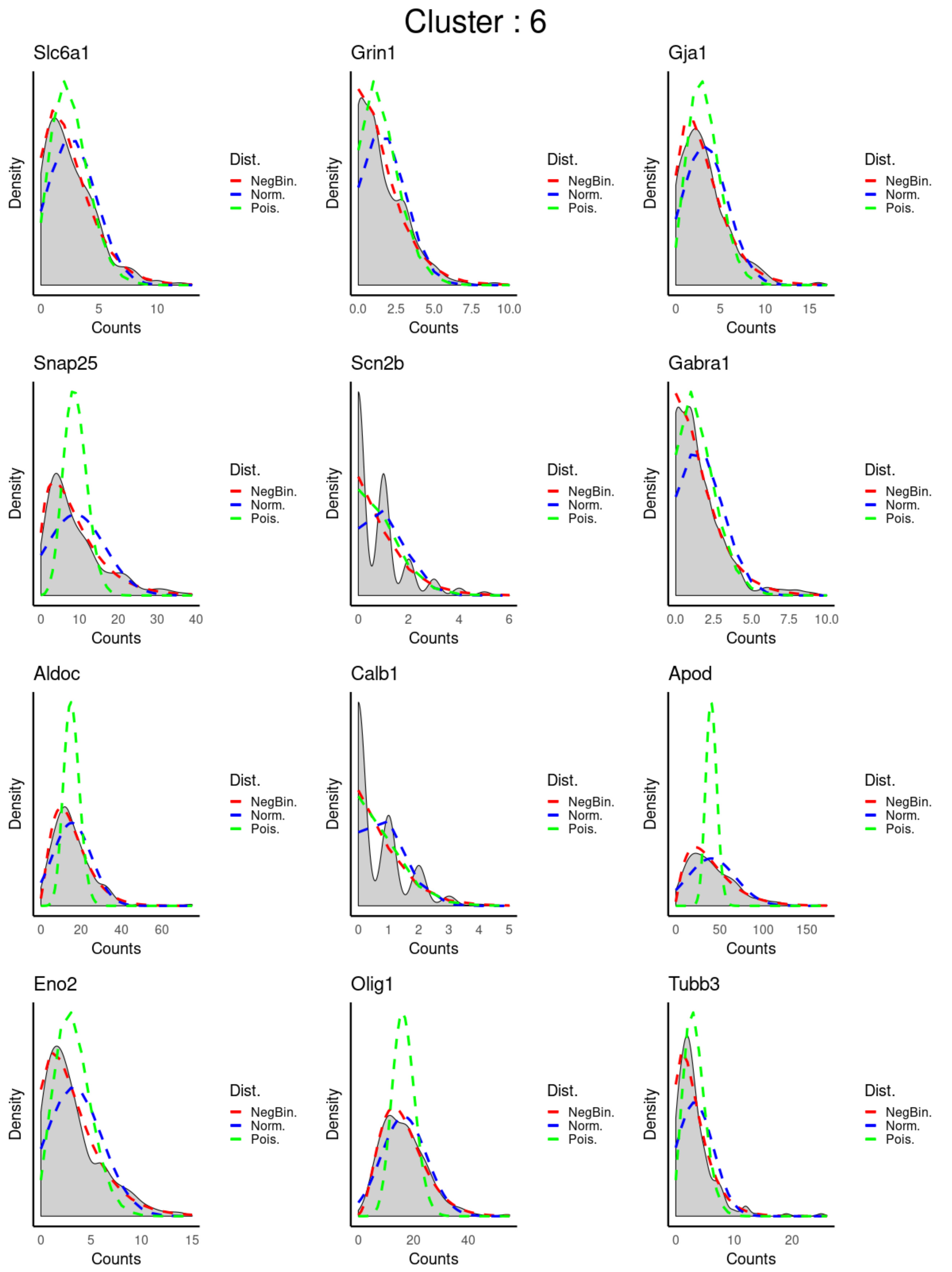

Supplementary Figure 28: Fitted vs. Empirical Distributions for Cluster 6. Gray - empirical distribution, Red - Negative Binomial, Green - Poisson, Blue - Normal

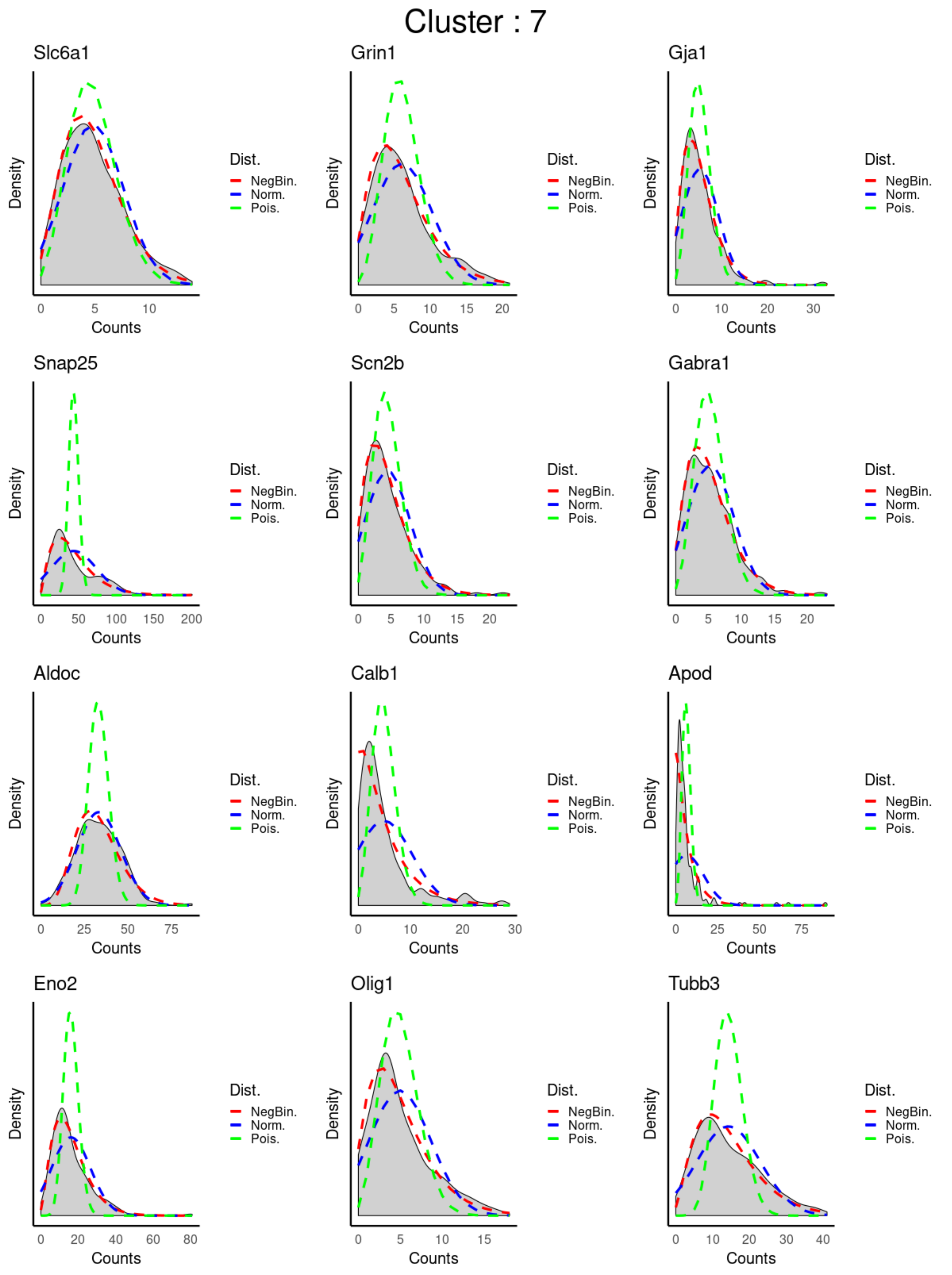

Supplementary Figure 29: Fitted vs. Empirical Distributions for Cluster 7. Gray - empirical distribution, Red - Negative Binomial, Green - Poisson, Blue - Normal

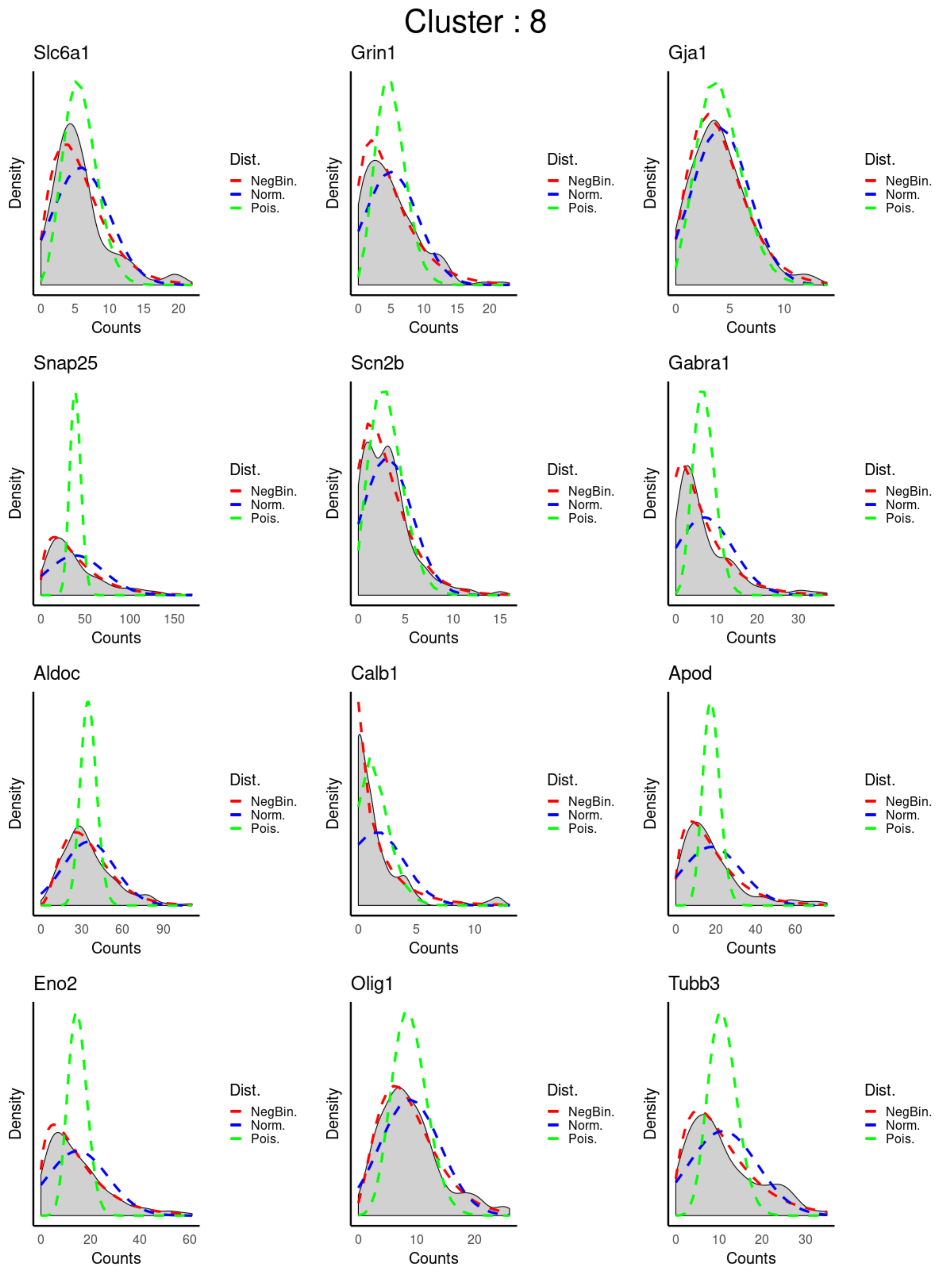

Supplementary Figure 30: Fitted vs. Empirical Distributions for Cluster 8. Gray - empirical distribution, Red - Negative Binomial, Green - Poisson, Blue - Normal

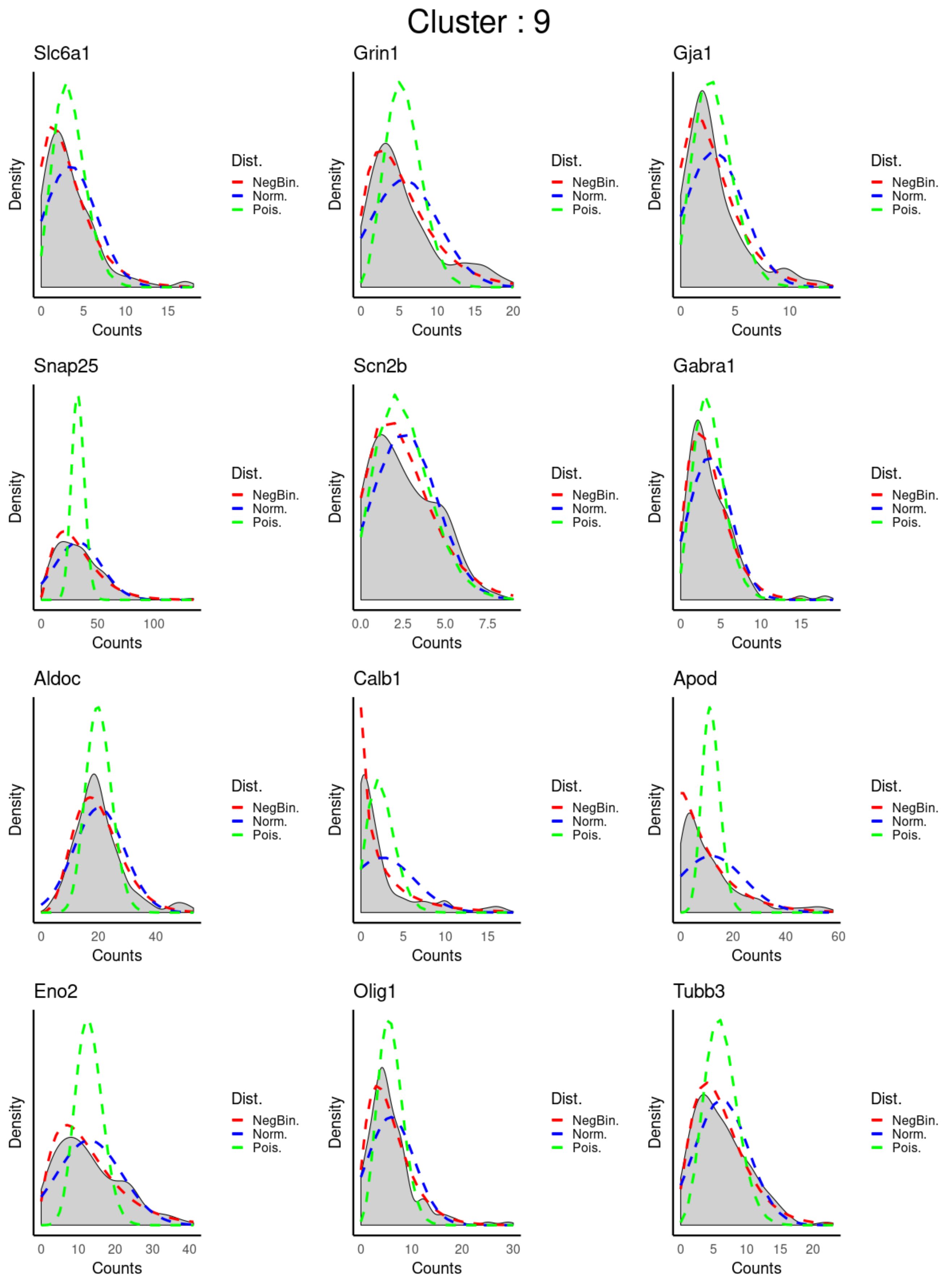

Supplementary Figure 31: Fitted vs. Empirical Distributions for Cluster 9. Gray - empirical distribution, Red - Negative Binomial, Green - Poisson, Blue - Normal

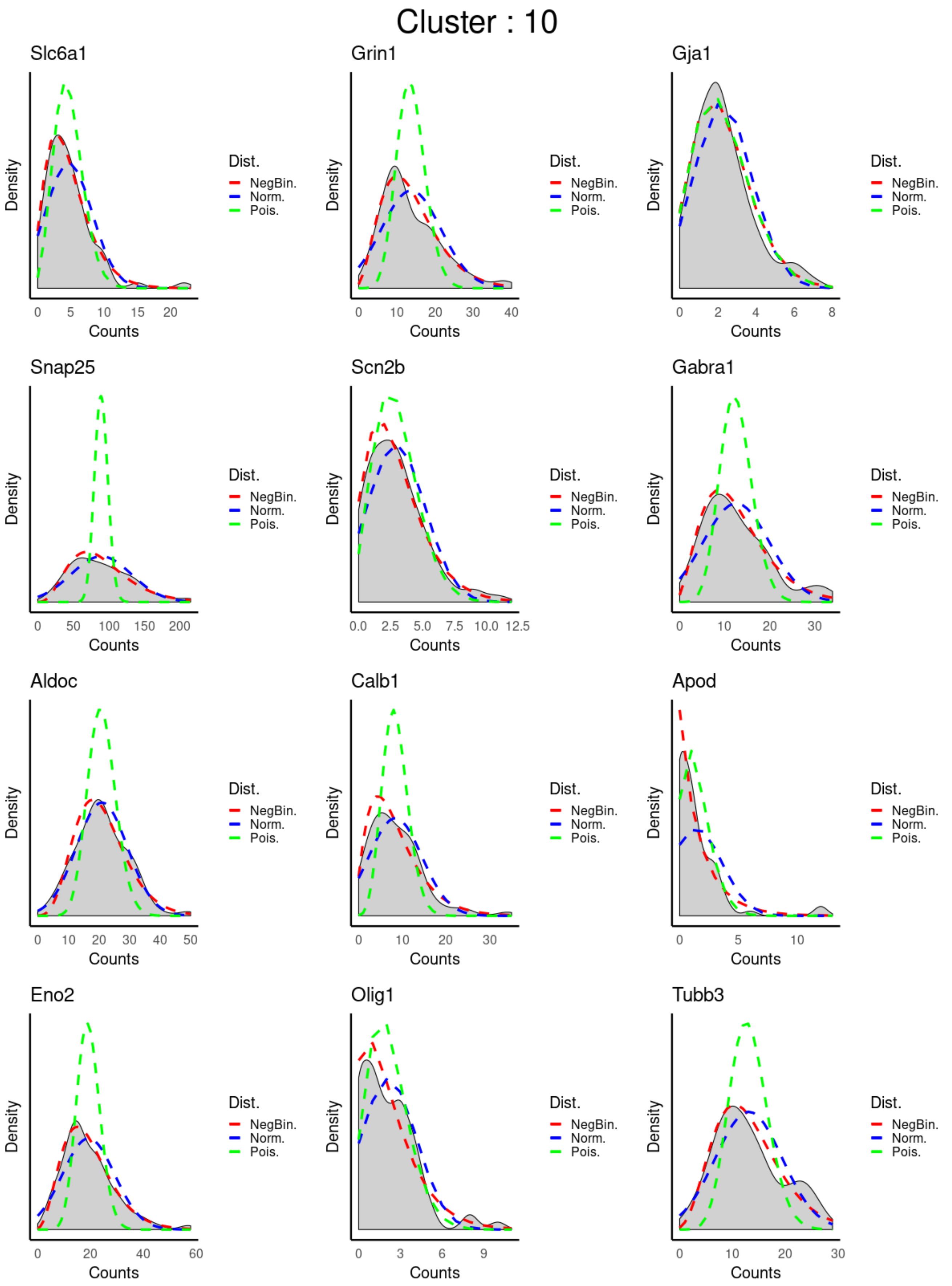

Supplementary Figure 32: Fitted vs. Empirical Distributions for Cluster 10. Gray - empirical distribution, Red - Negative Binomial, Green - Poisson, Blue - Normal

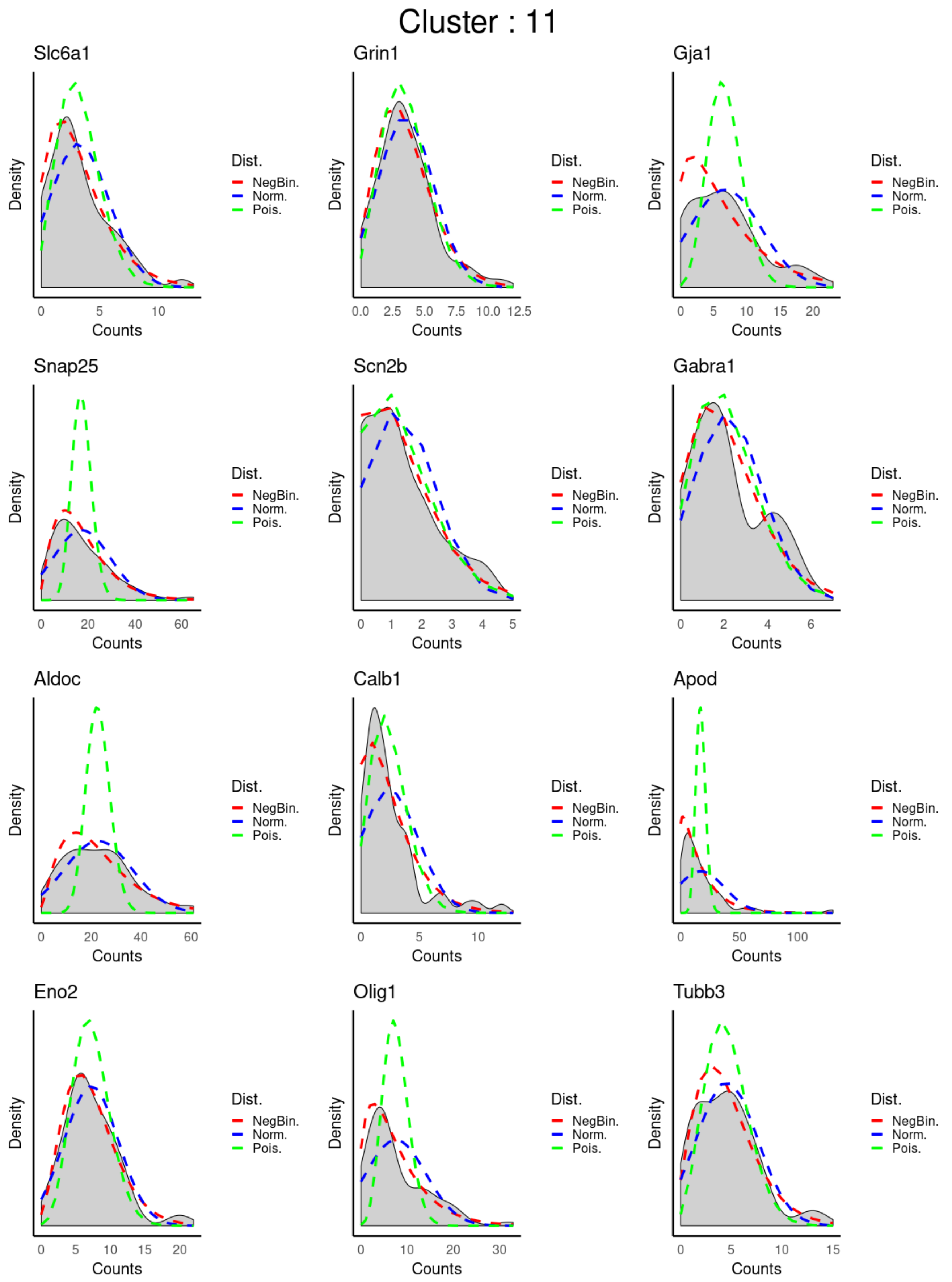

Supplementary Figure 33: Fitted vs. Empirical Distributions for Cluster 11. Gray - empirical distribution, Red - Negative Binomial, Green - Poisson, Blue - Normal

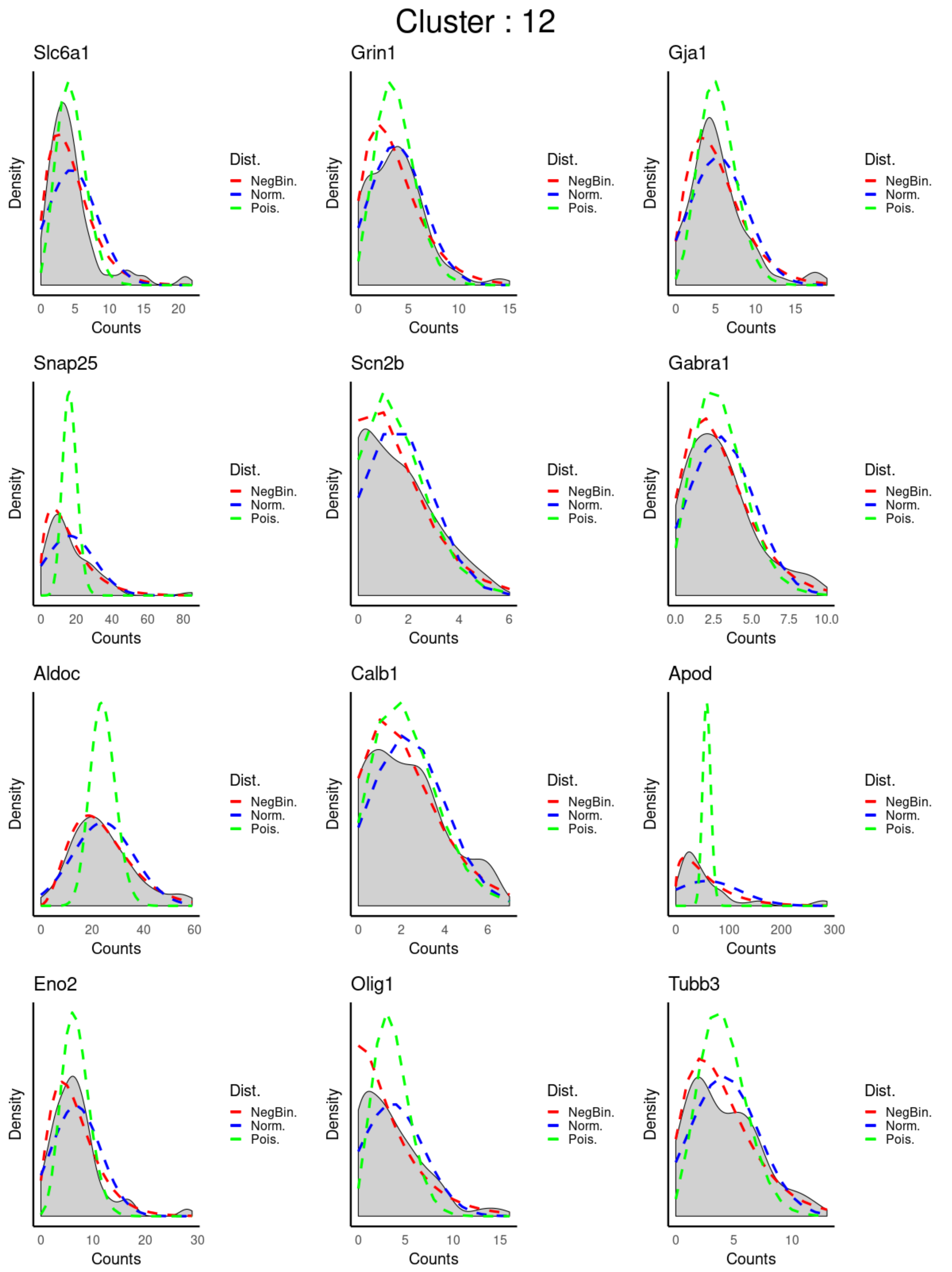

Supplementary Figure 34: Fitted vs. Empirical Distributions for Cluster 12. Gray - empirical distribution, Red - Negative Binomial, Green - Poisson, Blue - Normal

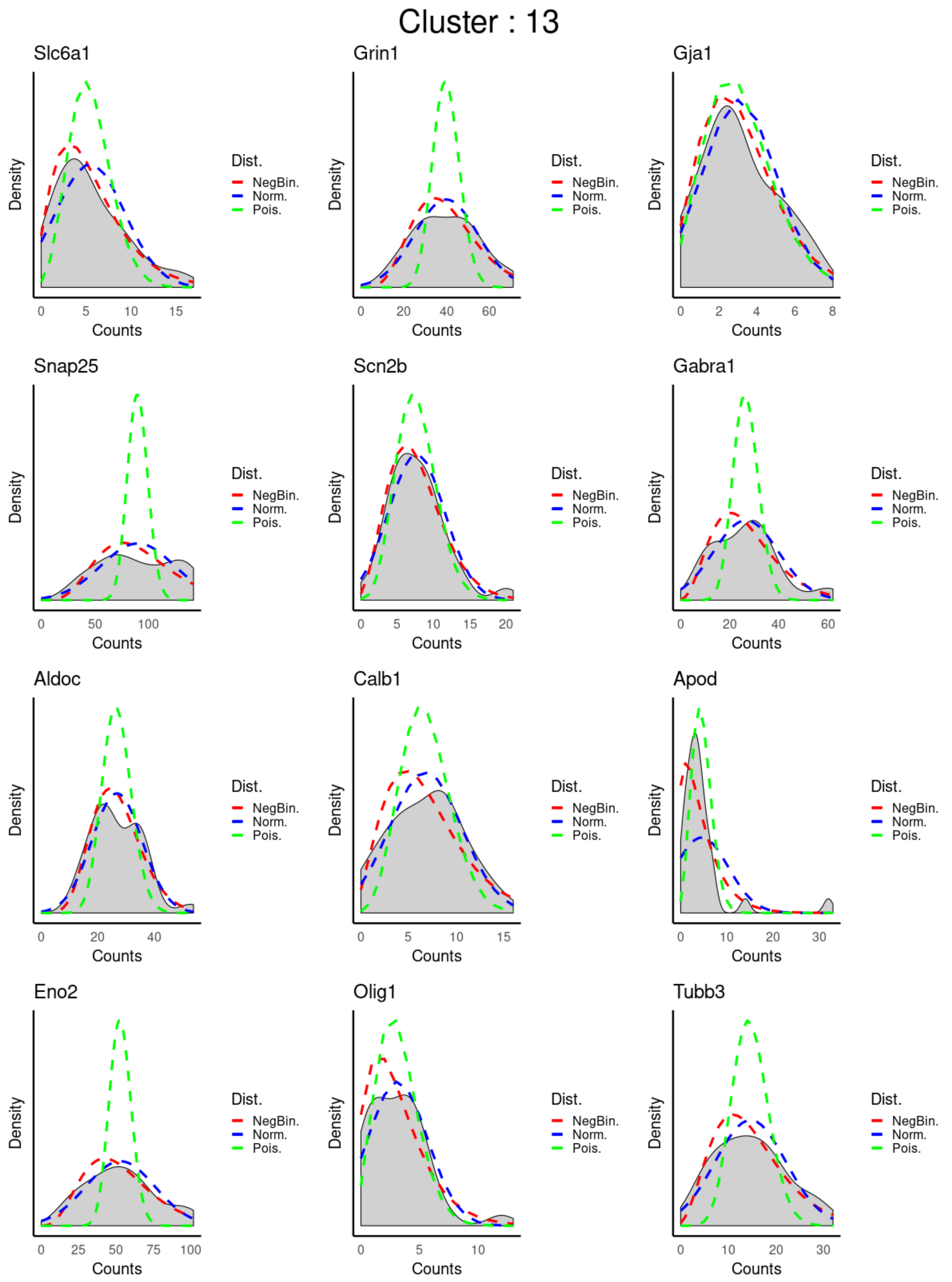

Supplementary Figure 35: Fitted vs. Empirical Distributions for Cluster 13. Gray - empirical distribution, Red - Negative Binomial, Green - Poisson, Blue - Normal

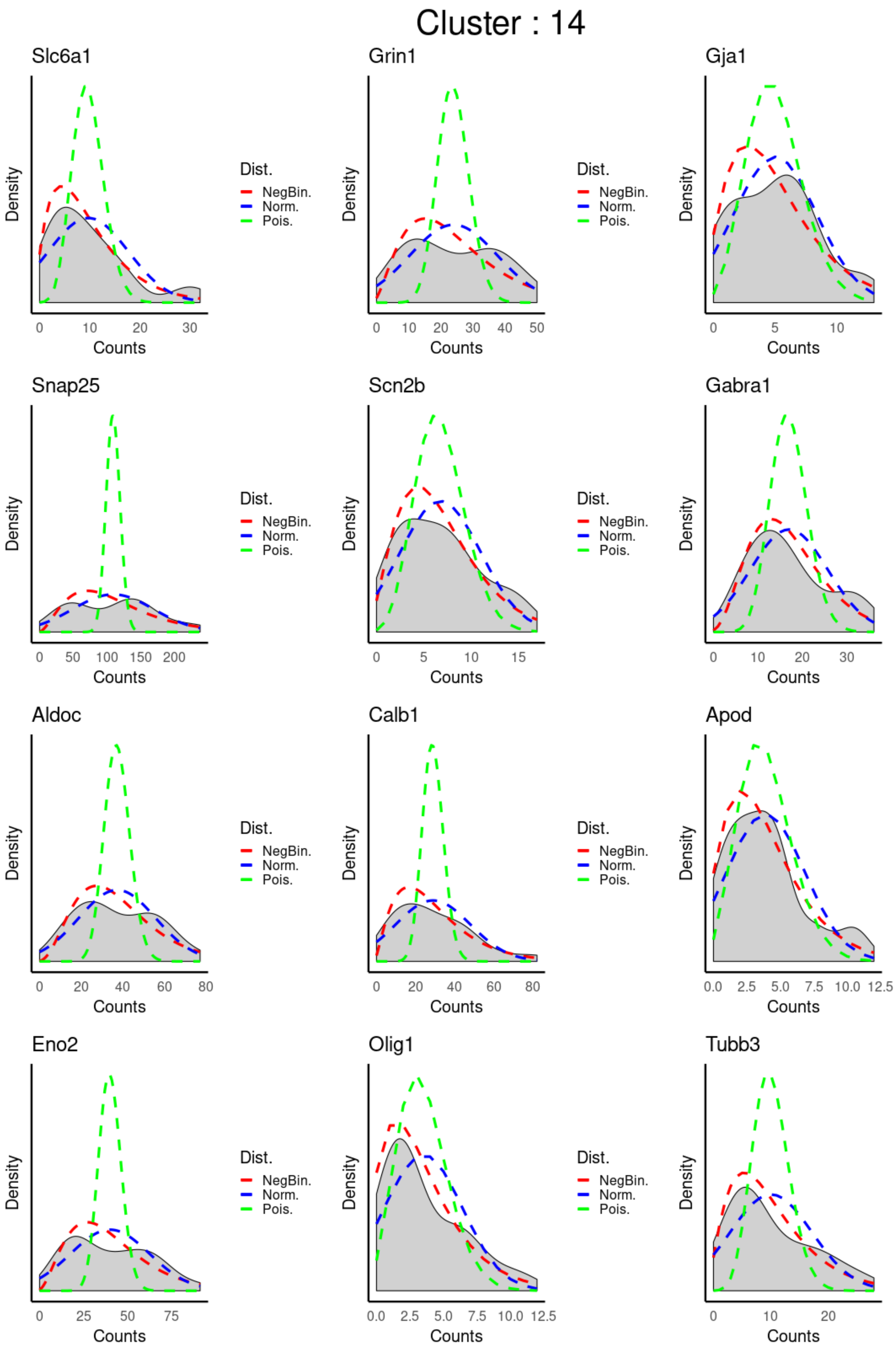

Supplementary Figure 36: Fitted vs. Empirical Distributions for Cluster 14. Gray - empirical distribution, Red - Negative Binomial, Green - Poisson, Blue - Normal

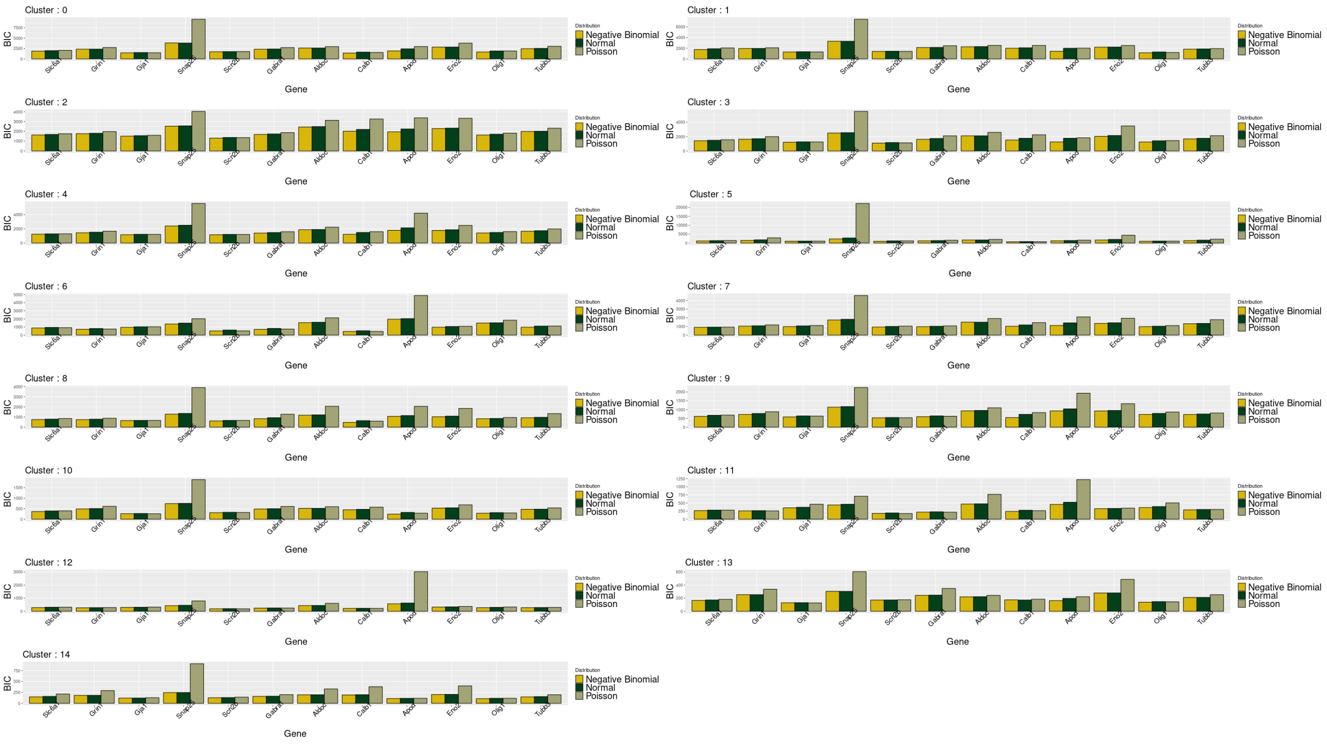

Supplementary Figure 37: BIC score for the different distributions fitted to each marker gene and across clusters.

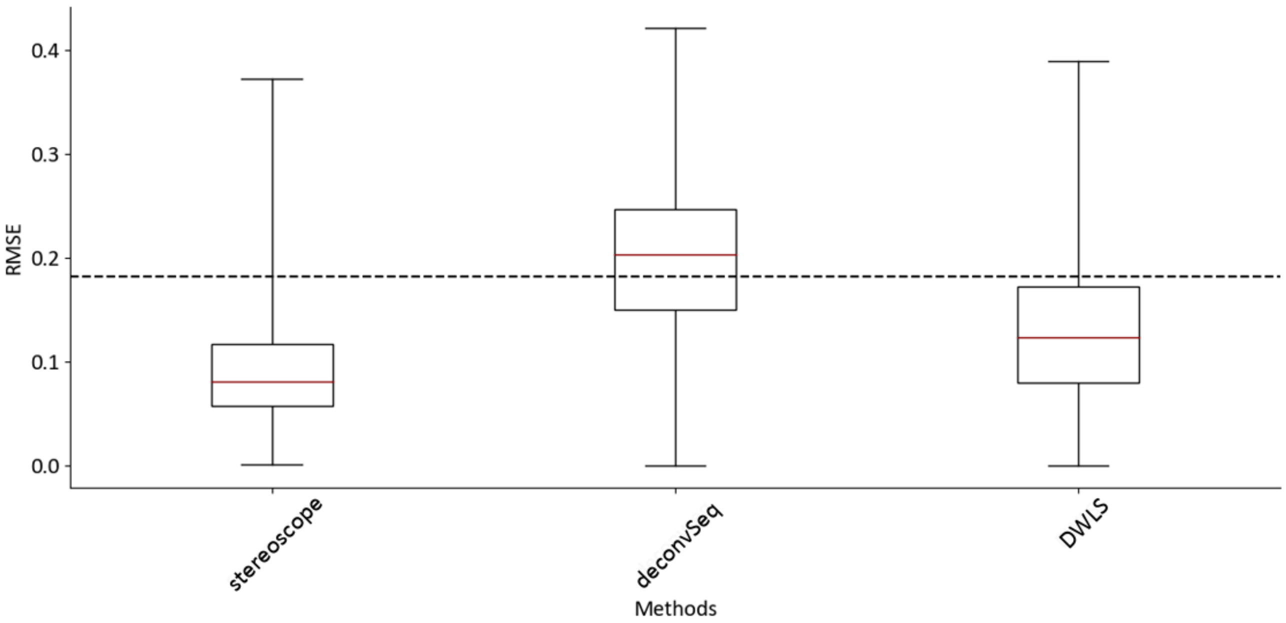

Supplementary Figure 38: Comparison between *stereoscope* and other methods designed to estimate proportions of cell types in bulk data with the help of single cell data. The boxes spans the range between the lower and upper quartiles of the data. The red line indicates the median. Whiskers show the full range. The dashed line indicates the average value from computing the RMSE from $n = 1000$ proportion estimate vectors (of each spatial location) generated from a Dirichlet distribution (concentration set to 1 for all types).

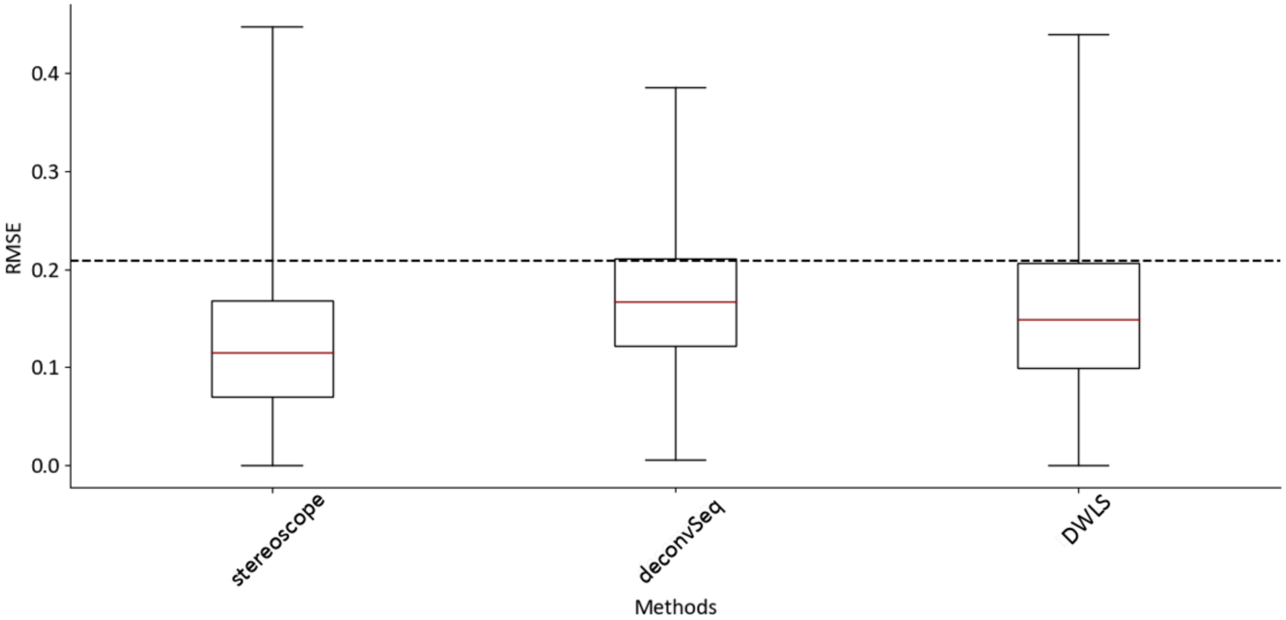

Supplementary Figure 39: Similar to Supplementary Figure 38 but for the synthetic data where cell density is set to range between $1-10$ cells.

Supplementary Tables

Data Set	Accession	Comment
Mouse Brain (ST1K)	`https://github.com/almaan/stereoscope/blob/master/data/mousebrain/mouse-st-data.zip` [1]	password : zNLXkYk3Q9znUseS
Mouse Brain (Visium)	`https://support.10xgenomics.com/spatial-gene-expression/datasets/1.0.0/V1_Adult_Mouse_Brain`	N/A
Mouse Brain Hippocampus (Slide-seq)	`https://singlecell.broadinstitute.org/single_cell/study/SCP354/slide-seq-study`	Puck : 180413_7
Mouse Brain Cerebellum (Slide-seq)	`https://singlecell.broadinstitute.org/single_cell/study/SCP354/slide-seq-study`	Puck : 180819_11
Mouse Brain Hippocampus (Single Cell)	`https://storage.googleapis.com/linnarsson-lab-loom/l1_hippocampus.loom`	N/A
Mouse Brain Cerebellum (Single Cell)	`https://storage.googleapis.com/linnarsson-lab-loom/l1_cerebellum.loom`	N/A
Developmental Heart (ST1K and Single Cell)	`https://www.spatialresearch.org/resources-published-datasets/doi-10-1016-j-cell-2019-11-025/`	N/A

Supplementary Table 1: Data access, links to all the data sets presented in the manuscript, additional information is given in the “Comments” columns when relevant.

	Cell Type	Number of Cells		Type	Number of Cells		Type	Number of Cells
**1**	Astrocytes_14	250	**20**	Neurons_15	250	**39**	Neurons_54	30
**2**	Astrocytes_40	250	**21**	Neurons_16	52	**40**	Neurons_58	48
**3**	Astrocytes_41	31	**22**	Neurons_17	250	**41**	Neurons_59	250
**4**	Astrocytes_42	250	**23**	Neurons_18	248	**42**	Neurons_60	250
**5**	Astrocytes_44	41	**24**	Neurons_19	55	**43**	Neurons_61	106
**6**	Blood_73	46	**25**	Neurons_20	38	**44**	Neurons_62	42
**7**	Ependymal_47	27	**26**	Neurons_21	250	**45**	Neurons_63	250
**8**	Excluded_30	30	**27**	Neurons_22	27	**46**	Oligos_0	250
**9**	Excluded_38	25	**28**	Neurons_23	249	**47**	Oligos_1	158
**10**	Excluded_44	56	**29**	Neurons_24	250	**48**	Oligos_14	101
**11**	Excluded_6	87	**30**	Neurons_25	250	**49**	Oligos_5	250
**12**	Immune_14	63	**31**	Neurons_26	250	**50**	Oligos_53	250
**13**	Immune_32	131	**32**	Neurons_27	250	**51**	Vascular_14	75
**14**	Immune_34	250	**33**	Neurons_28	250	**52**	Vascular_46	61
**15**	Immune_35	38	**34**	Neurons_30	26	**53**	Vascular_67	250
**16**	Neurons_10	35	**35**	Neurons_48	183	**54**	Vascular_68	250
**17**	Neurons_11	250	**36**	Neurons_49	25	**55**	Vascular_69	41
**18**	Neurons_12	250	**37**	Neurons_51	250	**56**	Vascular_70	33
**19**	Neurons_14	250	**38**	Neurons_52	241		**Total**	**8449**

Supplementary Table 2: Composition of the subsampled single cell mouse brain hippocampus data set.

	Cluster	Number of Cells		Cluster	Number of Cells
**1**	1	156	**19**	22	62
**2**	2	126	**20**	23	500
**3**	3	434	**21**	24	466
**4**	4	378	**22**	25	317
**5**	6	44	**23**	26	151
**6**	7	48	**24**	27	48
**7**	8	58	**25**	30	27
**8**	9	187	**26**	31	499
**9**	10	69	**27**	32	498
**10**	11	30	**28**	33	91
**11**	12	29	**29**	36	499
**12**	13	28	**30**	37	125
**13**	16	282	**31**	38	76
**14**	17	92	**32**	40	499
**15**	18	33	**33**	42	467
**16**	19	144	**34**	44	133
**17**	20	49	**35**	45	361
**18**	21	500		**Total**	7506

Supplementary Table 3: Composition of the subsampled single cell mouse brain cerebellum data set.

	Cell Type	Number of Cells
**1**	Atrial_cardiomyocytes	152
**2**	Capillary_endothelium	662
**3**	Cardiac_neural_crest_cells_Schwann_progenitor_...	75
**4**	Endothelium_pericytes_adventitia	127
**5**	Epicardial_cells	128
**6**	Epicardium-derived_cells	392
**7**	Erythrocytes_11	113
**8**	Erythrocytes_6	186
**9**	Fibroblast-like_cardiac_skeleton_connective_ti...	463
**10**	Fibroblast-like_larger_larger_vascular_develop...	150
**11**	Fibroblast-like_smaller_vascular_development	337
**12**	Immune_cells	76
**13**	Myoz2-enriched_cardiomyocytes	97
**14**	Smooth_muscle_cells_fibroblast-like	263
**15**	Ventricular_cardiomyocytes	496
	**Total**	**3717**

Supplementary Table 4: Composition of the single cell developmental heart data set.

	Cell Type	Number of Cells
**1**	Astrocyte	250
**2**	Astrocyte,Neurons	58
**3**	Astrocyte,Oligos	47
**4**	Blood	40
**5**	Immune	250
**6**	Neurons	250
**7**	Neurons,Cycling	94
**8**	Neurons,Oligos	33
**9**	Oligos	250
**10**	Vascular	250
	**Total**	**1522**

Supplementary Table 5: Composition of the *generation* and *validation* single cell data sets (the two share identical compositions).

Compared to.	*W*	*estimate*	*p-value*	*CI-upper*
deconvSeq	33148.0	-0.110118	6.571734e-107	-0.105184
DWLS	59784.0	-0.035190	2.970104e-77	-0.032272

Supplementary Table 6: Results from an one-sided paired Wilcoxon signed-rank test between ours (*stereoscope*) and other methods devised for bulk deconvolution aided by single cell data. In short, this tests whether the difference between the location-wise paired RMSE values are symmetrically distributed around zero, or if this distribution is skewed in favor of stereoscope. W is the test statistic, *estimate* is the estimated location of the difference between the estimates from *stereoscope* and the other methods. The p-value represents the probability of no assymetry existing between the methods. *CI-upper* is the upper 95% confidence level. Hence, a negative estimate with a significant p-value is to be interpreted in favor of stereoscope. The test was conducted using the *wilcox.test* function in R (v.3.5) with a total of $n = 1000$ synthetic data spots

Compared to.	*W*	*estimate*	*p-value*	*CI-upper*
deconvSeq	103377.0	-0.046208	1.176005e-48	-0.041459
DWLS	127372.0	-0.024165	2.110848e-32	-0.020732

Supplementary Table 7: Similar to Supplementary Table 6 but for the synthetic data where cell density is set to range between $1 - 10$ cells.

1 Supplementary Notes

1.1 Characterization of Spatial Expression Data

Our method assumes that gene expression data can be sufficiently modeled by a Negative Binomial (NB) distribution. This idea is by no means novel; several methods designed for analysis of either bulk or single cell RNA-seq data relies on variations of the very same assumption (e.g., edgeR, DeSeq2, ZINB-WaVE and sctransform) [3–6]. Spatial data generated from capture based technologies, like those discussed within this work, share several features with single cell data; the most obvious being how both are constituted of positive integer values representing the number of transcripts associated with a given observation (cell alternatively capture location). The experimental methods by which such spatial data is obtained nevertheless differ substantially from those of single cell data, and thus caution should be payed to extrapolation of assumptions between the data modalities. Aware of this, we here seek to justify our assumptions and present support for our spatial data being NB-distributed, as to ensure we're not working on false premises.

To briefly recapitulate some of the terminology and properties of the NB distribution: We let x_{sgc} denote the observable transcripts of a given gene (g), from a specific cell (c), at a given capture location (s). Our main assumption is that $x_{sgc} \sim \mathcal{NB}(., p_g)$, as a consequence of the additive property of NB distributions with shared second parameters (p_g), we therefore have that $x_{sg} = \sum_{c \in C_s} x_{sgc} \sim \mathcal{NB}(., p_g)$ as well.

There are however two prominent issues that must be addressed in the context of this quest, namely that: (1) x_{sgc} is not observed in any of the experimental platforms included in this study, and we are therefore limited to computational inferences of these values; (2) while we do observe x_{sg} (being entries of the count matrices), we only have *one* such observation per distribution that we wish to characterize; this sparsity prevents us from making any general statement regarding the distributions. Due to (1) we will focus our efforts on characterization of x_{sg} rather than x_{sgc}, to then consider support for the former being NB distributed as strongly implicating the latter having the same property. In order to circumvent (2), we will cluster the capture locations based on their gene expression profiles and assume that members within the same cluster (k) can be taken to have approximately the same first parameter (r_{kg}); meaning that $x_{sg}|s \in k = x_{kg} \sim \mathcal{NB}(r_{kg}, p_g)$. As a result of this simplification, we have multiple observations from respective distribution (x_{kg}) and are able evaluate how well the NB distribution approximates this.

For the purpose of this inquiry, we use the mb-V1 section (Visium) and a set of genes ($n = 12$) listed as markers for cell types within the brain (taken from `panglaodb.se`), these genes also exhibit varying spatial distributions, see Supplementary Figure 20. [2] We used the *Seurat* package in R to normalize and cluster our data, from this we obtained 15 clusters, shown in Figure 21. See Supplementary Section 1.1.1 for more details regarding the clustering. Next, within each cluster and for each marker gene, we fitted 3 different parametric distributions (Negative Binomial, Normal and Poisson) to the observed expression (using the R package *fitdistrplus*). The empirical and fitted distributions for each cluster and marker gene are visualized in Supplementary Figures 22-36. As can be seen in the aforementioned figures, the NB distribution (red) tends to provide a good approximation for the observed empirical distribution (gray). Some deviations and ill-fits are observed, but these are mainly confined to clusters with few members or where the specific gene's expression do not overlap very well with the cluster (in the spatial domain). For a more quantitative assessment of how well the different distributions were able to describe the data, we compared their respective BIC (*Bayesian information criterion*) values, once the distributions had been fitted to the data. For all marker genes, the NB distribution outperformed the alternative distributions, as shown in Supplementary Figure 37.

We believe these results support and speak in favor of our assumption that x_{sg} is well approximated by an NB distribution, hence also being affirmative of our model's design. Furthermore, from a theoretical standpoint it's also motivated to take the spatial data as NB-distributed; the NB distribution is often interpreted as an "overdispersed" Poisson distribution, which represents the number of iid events that occur in given interval of time or space - a definition that translates well to the capture of mRNA at specific locations in the spatial assays. If we were to accept this as sufficient support for our assumption regarding the sum x_{sg}, it's also motivated to assume that the respective constituents are NB-distributed, as this would indeed produce a new NB-distributed variable like that of x_{sg}.

All results related to the discussion regarding the assumption of spatial capture-based data being Negative Bino-

mial distribution, can be reproduced by running the script *test-NB.R* (found in the referenced github and Zenodo repositories), where the only input needed is the mb-V1 count file and a list of genes (the marker genes used) to be analyzed.

1.1.1 Seurat Clustering

To cluster the data, we followed the steps outlined in the Seurat Vignette [7], without any modifications (meaning, *resolution* = 0.8). In total 15 clusters were identified, as presented in Supplementary Figure 21.

1.2 Method Comparison

To compare and benchmark our method against alternative methods designed for deconvolution of bulk RNA-seq data using single cell RNA-seq data, we generated synthetic data according to the procedure outlined in Table 1 (Methods). The synthetic data generation produces sets with known proportion values, which can be used to quantitatively evaluate method performance. We conducted two comparative analyses:

- **Comparison 1, 10-30 cells per capture location**: For this comparison; cell density (cells per capture location) was set to $10 - 30$ cells during synthetic data generation. The intention was to generate data that resembled that obtained from the older ST (1k array) platform. Results are shown in Supplementary Figure 38 and Supplementary Table 6, where it can be seen how *stereoscope* outperforms the other methods.
- **Comparison 2, 1-10 cells per capture location**: For this comparison; cell density (cells per capture location) was set to $1 - 10$ cells during synthetic data generation. For this set the intetion was to generate data that resembled that produced by the Visium platform. Results are shown in Supplementary Figure 39 and Supplementary Table 7, *stereoscope* performs better than the alternative methods in this comparison as well.

1.3 Data : Mouse Brain

Single Cell
Single cell data was downloaded from *mousebrain.org*, where data from Hippocampus and Cerebellum were provided as loom-files containing a total of 29519 cells (hippocampus) and 27998 cells (cerebellum). As stated in the methods section: for the hippocampus data, the labels given as "Clusters" and "Class" were joined together in order to define more granular cell types; while for the cerebellum data we used the "Clusters" labels. Applying the subsampling scheme described in Methods, data sets consisting of 8449 (hippocampus) and 7506 (cerbellum) cells were assembled, the exact compositions of these sets are found in Supplementary Table 2 and 3.

ST/Visium
We analyzed two 100 micron array ST sections (mb-ST1 and mb-ST2), data can be accessed at the github page, original source in currently in press. We excluded all spots that were not covered by the tissue. We downloaded Visium (55 micron array) data from the website of 10x Genomics^{TM}, listed under "Support", "Spatial Gene Expression" and "Datasets", selecting the set listed as "Mouse Brain Section (Coronal)". [8] For the Visium data we only included spots under the tissue in our analysis.

Slide-seq
We analyzed two Slide-seq pucks: the puck visualized in Fig.1C (hippocampus) and one of the pucks used to generate Fig.2C (cerebellum) - figure numbers refer to the publication where the method was first presented. [1] The data was accessed from the "Single Cell Portal" provided by Broad Institute, pucks with ID 180413_7 (hippocampus) and 180819_11 (cerebellum) were downloaded. We used the files *MappedDGEForR.csv* and *MappedLocationsForR.csv*, associated with pucks of said IDs, to assemble an expression matrix with beads as rows and genes as columns. [9] We also replaced the original row names containing the barcode ids with each bead's spatial coordinates given as "[x_coordinate]x[y_coordinate]". All beads with non-zero total counts were used in the analysis.

1.4 Data : Developmental Heart

Both single cell and ST data for the analysis of the developmental heart are taken from the publication "*A spatiotemporal organ-wide gene expression and cell atlas of the developing human heart.*". [10] The complete set of

single cells was used, while only the 8 ST sections from PCW (Post Conceptional Week) 6.5 were used as spatial data. Supplementary Table 4 gives the specifics of the single cell data set.

1.5 Data : Synthetic Data

Single Cell
To generate synthetic single cell data, the data set originating from hippocampal tissue was downloaded from *mousebrain.org* (the same set as used for the analysis of the mouse brain) where the "Subclass" annotations were used as cell type identifiers. A generation and validation set of equal compositions (in terms of number of cells from each cell type) were generated, exact structure given in Supplementary Table 5.

Spatial Data
A total of 1000 spots were synthesized according to the procedure described in the Methods section. Only data from the top 500 highest expressed genes in the generation data set were used, hence a 1000×500 count matrix was generated. All synthetic data sets and the code used for the "synthesis" is available in the github repository of this paper, where also a tutorial to reproduce the results is presented.

1.6 Slide-seq analysis

With no histological image being provided for the Slide-seq data, we depict the obtained proportion estimates in a similar fashion to the ST data – each bead is represented by a circular marker where the alpha value is proportional to the estimated proportion for each cell type – but without a background tissue image. Due to the large number of beads present in the Slide-seq assays (and thus data points to visualize) the proportion estimates are multiplied by the scalar 0.6 in Fig.15, rendering a result which is easier to inspect and interpret. While Slide-seq provides high resolution, a one-to-one mapping between bead and cell is not guaranteed, given how beads tend to have between 1-3 cells contributing to them. [1] We therefore do not apply "hard" cluster assignments (i.e., assigning each bead to the type with highest associated proportion estimate) but rather use the proportion estimates. No scaling within cell type nor section is performed upon visualization.

Our main reason for including the cerebellum Slide-seq data was to assess whether we obtained the same number of cell types distributed across the beads as reported in the original Slide-seq study. The authors report that for the 7 Cerebellum pucks approximately $65.8 \pm 1.4\%$ of the beads are matched to a single cell type while $32.6 \pm 1.2\%$ mapped to two cell types (numbers reported as mean $\pm$ standard deviation).

To compare our results with those obtained in the Slide-seq study, we implemented an approach similar to what the Slide-seq authors used when calling the number of cell types assigned to a bead; in our case a cell type was "confidently assigned" to a bead if the proportion value of a cell type was greater than half the L2 norm of a bead's proportion vector. By doing so we could observe how 60.2% and 37.6% of beads had one respectively two cell types confidently assigned to them, see Figure 18. While these values both fall outside of the reported error range, it should be noted that we used a different single cell data set with a larger number of cell types; it's therefore plausible to suggest that what was called as contribution from one cell type in the Slide-seq study might in some cases (since we are operating at a higher granularity) be matched to two types in our data - explaining the slight discrepancies between the reported values.

References

[1] Samuel G. Rodriques, Robert R. Stickels, Aleksandrina Goeva, Carly A. Martin, Evan Murray, Charles R. Vanderburg, Joshua Welch, Linlin M. Chen, Fei Chen, and Evan Z. Macosko. Slide-seq: A scalable technology for measuring genome-wide expression at high spatial resolution. *Science*, 363(6434):1463–1467, March 2019.

[2] Oscar Franzén, Li-Ming Gan, Johan L M Björkegren PanglaoDB: a web server for exploration of mouse and human single-cell RNA sequencing data *Database* 2019(2019), April 2019 doi : 10.1093/database/baz046

[3] M. D. Robinson, D. J. McCarthy, and G. K. Smyth. edgeR: a bioconductor package for differential expression analysis of digital gene expression data. *Bioinformatics*, 26(1):139–140, November 2009.

[4] Michael Love, Wolfgang Huber, and Simon Anders. Moderated estimation of fold change and dispersion for RNA-seq data with DESeq2. *Genome Biology*, 15(12), December 2014.

[5] Davide Risso and Fanny Perraudeau and Svetlana Gribkova and Sandrine Dudoit and Jean-Philippe Vert A general and flexible method for signal extraction from single-cell RNA-seq data *Nature Communications* 9(1), December 2019 doi : 10.1038/s41467-017-02554-5

[6] Christoph Hafemeister and Rahul Satija Normalization and variance stabilization of single-cell RNA-seq data using regularized negative binomial regression *Genome Biology* 20(1), January 2018 doi : 10.1038/s41467-017-02554-5

[7] Seurat Documentation Seurat - Guided Clustering Tutorial `https://satijalab.org/seurat/v3.1/pbmc3k_tutorial.html`. (Accessed: 2020-07-20).

[8] 10x Genomics^{TM}. Spatial gene expression : Visualize Gene Expression within Tissue Organization `https://www.10xgenomics.com/solutions/spatial-gene-expression/`. (Accessed: 11.12.2019).

[9] Broad Single Cell Portal Study: Slide-seq study `https://singlecell.broadinstitute.org/single_cell/study/SCP354/slide-seq-study`. (Accessed: 11.12.2019).

[10] Michaela Asp, Stefania Giacomello, Daniel Fürth, Johan Reimegård, Eva Wärdell, Joaquin Custodio, Fredrik Salmén, Erik Sundström, Elisabet Åkesson, Magda Bienko, Agneta Månsson-Broberg, Patrik L. Ståhl, Christer Sylvén, and Joakim Lundeberg. A spatiotemporal organ-wide gene expression and cell atlas of the developing human heart. *Cell*, 179(7):1647-1660, 12 12 2019.
